# Expression of factor XIII originating from synovial fibroblasts and macrophages induced by interleukin-6 signaling

**DOI:** 10.1186/s41232-022-00252-4

**Published:** 2023-01-06

**Authors:** Hirofumi Watanabe, Sho Mokuda, Tadahiro Tokunaga, Hiroki Kohno, Michinori Ishitoku, Kei Araki, Tomohiro Sugimoto, Yusuke Yoshida, Toshihiro Yamamoto, Mayuko Matsumoto, Junya Masumoto, Shintaro Hirata, Eiji Sugiyama

**Affiliations:** 1 Department of Clinical Immunology and Rheumatology, Hiroshima University Hospital, 1-2-3, Kasumi, Minami-ku, Hiroshima, 734-8551 Japan; 2 Department of Pathology, Ehime University Proteo-Science Center and Graduate School of Medicine, Shitsukawa, Toon, Ehime, 791-0295 Japan

**Keywords:** Rheumatoid arthritis, Blood coagulation factor XIII, Monocyte-derived macrophages, Fibroblast-like synoviocytes (FLS), Interleukin-6

## Abstract

**Background:**

Blood coagulation factor XIII (FXIII) promotes cross-linking between fibrin molecules at the final stage of the blood coagulation cascade. However, its expression in cells or tissues and function, particularly factor XIII subunit B (FXIII-B), remains controversial. Hemorrhagic FXIII deficiency following anti-interleukin-6 (IL-6) receptor antibody treatment has been reported in patients with rheumatoid arthritis (RA). Patients receiving this biologics have reduced FXIII activity when compared to the activity in those treated with other biologics. The relationship between pro-inflammatory cytokines and FXIII expression remains unknown.

**Methods:**

To investigate the expression pattern of FXIII in synovial tissues, immunohistochemistry, RT-qPCR, and western blotting were performed. FXIII-A expressed monocyte-derived macrophages were treated with recombinant IL-6 and anti-IL-6 receptor antibody. RNA sequencing of FXIII-B-overexpressing cells was performed to clarify the function of FXIII-B.

**Results:**

The immunohistochemical analysis of synovial tissues revealed that factor XIII subunit A (FXIII-A) was expressed in M2 macrophages, and FXIII-B was expressed in fibroblast-like synoviocytes. IL-6 stimulation upregulated FXIII-A expression in IL-4-induced monocyte-derived macrophages, and the anti-IL-6 receptor antibody suppressed FXIII-A expression. FXIII-B was more abundantly secreted in the supernatant of fibroblast-like synoviocytes compared with that of other cells. RNA sequencing showed that FXIII-B elevated the expression of genes associated with anti-apoptotic molecules and chemokines.

**Conclusions:**

Our findings highlight that synovial tissue is one of the sources of FXIII production. We also have demonstrated IL-6-dependent FXIII-A expression and the novel potential functions of FXIII-B.

**Supplementary Information:**

The online version contains supplementary material available at 10.1186/s41232-022-00252-4.

## Background

Blood coagulation factor XIII (FXIII), also known as a fibrin-stabilizing factor, promotes cross-linking between fibrin molecules at the final stage of the blood coagulation cascade. It maintains complete hemostasis and promotes wound healing [1–4]. In plasma, FXIII is heterotetrametric, consisting of two A subunits (FXIII-A) and two B subunits (FXIII-B). FXIII-A exhibits enzymatic activity and stabilizes fibrin. Moreover, FXIII-B is a transport carrier for FXIII-A and plays an auxiliary role in fibrin stabilization [2].

Rheumatoid arthritis (RA) is a chronic inflammatory disease characterized by synovial hyperplasia, inflammatory cell infiltration, and irreversible cartilage and bone destruction [5]. In 2013, our research group first reported low FXIII activity in patients treated with tocilizumab, a monoclonal antibody against the interleukin-6 receptor (IL-6R), which is one of the treatment strategies against RA [6]. Subsequent studies also revealed reduced plasma concentrations of both FXIII-A and FXIII-B in patients with RA treated with tocilizumab [7]. The relationship between pro-inflammatory cytokines and FXIII expression also remains unknown.

FXIII-A is expressed in monocytes [8, 9], macrophages [9], and megakaryocytes [10], while FXIII-B is produced in hepatocytes [11]. Conflicting immunohistochemistry results have shown that vascular endothelium, renal tubules, and fibroblasts express FXIII-B, not hepatocytes [12]. There are no studies that have evaluated the FXIII expression in synovial tissues. Herein, we aimed to identify tissues expressing FXIII-A and FXIII-B and evaluate these expression profiles, regulatory mechanisms induced by cytokines, and specific functions in synovial tissues.

## Methods

### Ethics

The present study was approved by the Clinical Ethics Committees of Hiroshima University Hospital and Dohgo Spa Hospital (approval number: E-668; approval date: 01/02/2017). The methods were performed in accordance with the approved guidelines. After obtaining written informed consent, synovial tissues were collected from patients with osteoarthritis (OA) and those with RA who fulfilled the classification criteria of the American College of Rheumatology 1987 and underwent total joint replacement or synovectomy.

### Preparation of peripheral blood mononuclear cells and fibroblast-like synoviocytes

All blood donors provided written informed consent to participate in the study. The procedure was approved by the Ethics Committee of Hiroshima University. Peripheral blood mononuclear cells (PBMCs) from healthy donors were separated using Lympholyte®-H density gradient centrifugation (Cedarlane, Burlington, NC, USA). For the preparation of fibroblast-like synoviocytes (FLS), synovial tissues from patients with RA were minced with 1 mg/mL ROCHE collagenase/dispase (Sigma-Aldrich, Tokyo, Japan) in phosphate-buffered saline (pH 7.2) for 1 h at 37 °C, filtered, and washed. The synovial cells were diluted and cultured. During culture, the supernatant was replaced to remove non-adherent cells. Adherent FLS were split at a ratio of 1:3 when 80 % confluent and passaged. FLS were used for experiments at passages 3–5.

### Immunohistochemistry staining for FFPE synovial tissues

Formalin-fixed paraffin-embedded (FFPE) synovial tissues were used for immunohistochemistry (IHC) analysis. The sections were deparaffinized and dehydrated, followed by microwaving in tris-ethylenediaminetetraacetic acid buffer (pH 9.0) for antigen retrieval. For enzymatic IHC staining, the sections were stained with primary antibodies and subsequently with alkaline-phosphatase-conjugated secondary antibodies. The following antibodies were used: anti-human FXIII-A antibody (mouse monoclonal, clone 2D2B2, Proteintech, Tokyo, Japan), anti-human FXIII-B antibody (rabbit polyclonal, Genetex, Irvine, CA, USA), Histofine Simple Stain AP Anti-Mouse (Nichirei Biosciences, Tokyo, Japan), and Histofine Simple Stain AP Anti-Rabbit (Nichirei Biosciences). The slides were incubated with ImmPACT Vector Red (Vector Laboratories, Newark, CA, USA) and counterstained with hematoxylin solution-modified Acc. for Gill II (Sigma-Aldrich). Sections were imaged using a Nikon (Tokyo, Japan) Eclipse Si. For fluorescent IHC staining, sections were subjected to antigen retrieval, as described above, and stained with primary antibodies. Double immunofluorescence labeling was performed by sequential incubation with Alexa Fluor 405-conjugated and Alexa Fluor 647-conjugated secondary antibodies. The following antibodies were used: anti-human FXIII-A antibody (mouse monoclonal, clone 2D2B2, Proteintech), anti-human FXIII-B rabbit pAb (rabbit polyclonal, Genetex), anti-human Cadherin11 (CDH11) antibody (goat polyclonal, R&D Systems Inc., Minneapolis, USA), anti-human CD68 (rabbit polyclonal, Proteintech), and anti-human CD14 antibody (rabbit polyclonal, Proteintech). Anti-human CD163 antibody (rabbit polyclonal, Proteintech), anti-human CD4 antibody (goat polyclonal, R&D Systems Inc.), anti-human CD8 antibody (rabbit monoclonal, R&D Systems Inc.), anti-human CD19 antibody (rabbit polyclonal, Proteintech), Alexa Fluor 405-conjugated anti-goat immunoglobulin G (IgG) antibodies (host: donkey, Abcam, Cambridge, UK), Alexa Fluor 405-conjugated anti-mouse IgG antibodies (host: donkey, Abcam), Alexa Fluor 647-conjugated anti-mouse IgG antibodies (host: donkey, Jackson ImmunoResearch, West Grove, PA, USA), and Alexa Fluor 647-conjugated anti-rabbit IgG antibodies (host: donkey, Jackson ImmunoResearch). Slides were quenched with the Vector TrueVIEW Autofluorescence Quenching Kit (Vector Laboratories). Nuclear staining was performed using acridine orange (Abcam). Subsequently, the sample glasses were enclosed on glass slides with VECTASHIELD Vibrance Antifade Mounting Medium (Vector Laboratories). Fluorescent images were obtained using a digital microscope VHX-7000 (KEYENCE, Osaka, Japan).

To counting the number of cells under FXIII-A or FXIII-B single fluorescent staining, the following analyses were performed: (1) lining and sublining layers were distinguished by referring to hematoxylin and eosin (HE) staining images, (2) the number of nuclear staining cells were considered the total cell count, and (3) the number of cells expressing FXIII-A or FXIII-B were counted and the ratio to the total cell count calculated. Subsequently, to count the number of cells in FXIII-A dual florescent staining, the following analyses were performed: (1) the number of FXIII-A-positive cells was considered the total cell count and (2) the number of cells expressing both FXIII-A and cluster of differentiation markers (double positive cells) were counted and the ratio to the total cell count was calculated. Lastly, to count the number of cells in FXIII-B dual florescent staining, the following analyses were performed: (1) lining and sublining layers were distinguished by refer to the HE staining images, (2) the number of FXIII-B-positive cells in the two locations above was considered the total cell count, and (2) the number of cells expressing both FXIII-B and CDH11 (double positive cells) was counted in two sites and the ratio to the total cell count calculated.

### Adherent cell culture

Primary FLS were cultured in Dulbecco’s modified Eagle’s medium (DMEM) (FUJIFILM Wako Pure Chemical Co., Osaka, Japan) supplemented with 10 % fetal bovine serum (FBS) (Sigma-Aldrich) and penicillin/streptomycin (FUJIFILM Wako Pure Chemical Co.). FLS were stimulated with or without recombinant human IL-6 and recombinant human soluble IL-6 receptor alpha (sIL-6Rα) (BioLegend). Immortalized rheumatoid fibroblast-like synoviocytes (MH7A cells) were obtained from KISSEI Pharmaceutical Co., Ltd. (Matsumoto, Japan). MH7A cells were cultured in Roswell Park Memorial Institute 1640 (RPMI 1640) medium (FUJIFILM Wako Pure Chemical Co.) supplemented with 10 % FBS and penicillin/streptomycin. HEK293T cells, which are immortalized human embryonic kidney cells, were purchased from ATCC (Manassas, VA, USA) and cultured in DMEM with 10 % FBS and penicillin/streptomycin. The cells were incubated at 37 °C under a 5 % CO_2_ condition.

### Monocyte separation and macrophage generation

PBMCs obtained as described above were separated using MACS microbeads (Pan Monocyte Isolation Kit; Miltenyi Biotec, Bergisch Gladbach, Germany), according to the manufacturer’s protocols. Based on morphology, the monocyte purity varied in the range of 95–98 %. Monocyte-derived macrophages were obtained by culturing monocytes in RPMI 1640 (FUJIFILM Wako Pure Chemical Co.) supplemented with 10 % FBS (Sigma-Aldrich), penicillin/streptomycin (FUJIFILM Wako Pure Chemical Co.), and 25 ng/mL macrophage colony-stimulating factor (M-CSF) (BioLegend, San Diego, CA, USA) for 5 days. M1 macrophages were generated using 20 ng/mL recombinant human interferon (IFN)-γ (PeproTech, Rocky Hill, NJ, USA) and 100 ng/mL lipopolysaccharides (LPS) (FUJIFILM Wako Pure Chemical Co.) for 24 h. M2 macrophages were generated using 20 ng/mL recombinant human interleukin (IL)-4 (R&D Systems Inc.) or 20 ng/ml recombinant human IL-10 (BioLegend) for 24 h. M0 macrophages were cultured in the medium and left untreated. Macrophages were stimulated with or without 20 ng/ml recombinant human IL-6 (BioLegend), 200 ng/ml anti-human IL-6R alpha antibody (mouse monoclonal, clone #17506, R&D Systems Inc.), or 200 ng/ml mouse IgG1 isotype control (mouse monoclonal, clone #11711, R&D Systems Inc.).

### Preparation of CD14-positive synovial macrophages

To prepare synovial macrophages, synovial tissues from patients with RA were treated as noted previously. Minced and filtered synovial cells, including FLS, synovial macrophages, and lymphocytes, were cultured, and non-adherent cells removed and used in the experiment at passages 1–2. Harvested cells were separated using MACS microbeads (Pan Monocyte Isolation Kit, Miltenyi Biotec), according to the manufacturer’s protocols. CD14-positive synovial macrophages were cultured using RPMI 1640 supplemented with 10 % FBS and penicillin/streptomycin and then stimulated with or without 20 ng/ml recombinant human IL-6 (BioLegend).

### Small interfering RNA transfection experiments for monocyte-derived macrophages and MH7A cell line

Monocyte-derived macrophages were transfected with small interfering RNA (siRNA) at 20 pmol using Lipofectamine RNAiMAX (Thermo Fisher Scientific, Waltham, MA, USA), according to the manufacturer’s protocol. MH7A cells were also transfected with siRNA at 10 pmol. The siRNA against Signal Transducers and Activator of Transcription 1 (STAT1) (FlexiTube siRNA, SI04950960), F13B (FlexiTube siRNA, SI03051202), and negative control (Allstar Negative Control siRNA) were purchased from QIAGEN (Shanghai, China).

### In vitro transcribed mRNA transfection into primary cultured FLS

Plasmids coding for FXIII-B (NM_001994.3) or enhanced green fluorescent protein (EGFP) were constructed by inserting full-length cDNA into pcDNA3-A(124) vectors, and in vitro transcribed (IVT) mRNA was prepared as previously described [13]. The insert fragment of the HA-tagged *F13B* open reading frame was purchased from GenScript (Tokyo, Japan). Empty pcDNA3-A(124) vector was also used for the preparation of mock IVT mRNA.

IVT mRNA was transfected into cells using Lipofectamine MessengerMAX (Thermo Fisher Scientific), according to the manufacturer’s instructions. Briefly, mRNA was mixed with the Lipofectamine reagent at a ratio of 1:3 (pmol mRNA: μL Lipofectamine) in serum-free DMEM. Complex formation was performed for 20 min at 25 °C. At 24 h after transfection, both cells and their culture supernatants were harvested for subsequent experiments.

### Luciferase assay

For the luciferase assay, a reporter plasmid was constructed using custom gene products (Genscript, Piscataway, NJ, USA). The pGL4.16 vector was purchased from Promega (Madison, WI, USA). The pGL4-F13A1p vector was constructed by inserting the human *F13A1* promoter region (from −783 to + 68 base pairs [bp] relative to the transcription start site [TSS]) into the pGL4.16 vector.

HEK293T cells were transfected with plasmid DNA using ScreenFect A plus (FUJIFILM Wako Pure Chemical Co.) according to the manufacturer’s instructions. After transfection of luciferase reporter plasmids, the cells were incubated for 24 h. The cells were stimulated with or without 20 ng/ml recombinant human IL-6 plus 20 ng/ml recombinant human sIL-6Rα (BioLegend) for 6 h before harvesting. Cells were lysed and measured using Dual-Glo Luciferase Assay System (Promega) in a 96-well plate. The luminescence intensities were measured using a SpectraMax iD3 system (Molecular Devices, San Jose, CA, USA).

### Western blotting

Proteins were harvested from the intracellular and culture supernatants. Before collection, MH7A cells, FLS, monocytes, and macrophages were washed with phosphate-buffered saline. The cells were dissolved in radioimmunoprecipitation assay (RIPA) buffer. For the supernatant protein assay, the last 24 h of incubation was performed in a serum-free medium. Cell supernatants for FLS, MH7A, HEK293T, and macrophages were suspended in acetone and kept at –20 °C for 1 h. The samples were then centrifuged for 10 min at 15,000 × *g* at 20–25°C. Protein precipitates were dried for 30 min and dissolved in RIPA buffer. Proteins were processed using a SuperSep Ace 15 % precast gel (FUJIFILM Wako Pure Chemical Co.) and transferred onto a polyvinylidene fluoride membrane. The membranes were probed with anti-human β-actin antibody (0.1 μg/mL, mouse monoclonal, clone AC-15, Sigma-Aldrich), anti-human FXIII-A antibody (0.025 μg/mL, mouse monoclonal, Proteintech), anti-human FXIII-B antibody (0.1 μg/mL, rabbit polyclonal, Sigma-Aldrich), and anti-HA-tag antibody (0.2 μg/mL, goat polyclonal, Genscript). Horseradish peroxidase-conjugated secondary antibodies (Jackson ImmunoResearch) were then added. Horseradish peroxidase activity was detected using ECL prime reagents (Cytiva, Tokyo, Japan), followed by imaging using the Image Quant LAS 500 (Cytiva) system.

### RNA isolation and reverse transcription-quantitative PCR

The cultured cells and mashed whole synovial tissues were dissolved in TRIzol (Life Technologies, Carlsbad, CA, USA), and RNA was extracted using Direct-zol RNA MicroPrep (Zymo Research, Orange, CA, USA), according to the manufacturer’s protocol. The total RNA (100 ng) was reverse-transcribed into cDNA using a PrimeScript RT Reagent Kit with gDNA Eraser (Takara Bio, Kusatsu, Japan). Reverse transcription-quantitative polymerase chain reaction (RT-qPCR) was performed using Brilliant III ultrafast SYBR Green qPCR Master Mix (Agilent Technologies, Santa Clara, CA, USA) on a CFX Connect Real-Time PCR Detection System (Bio-Rad Laboratories, Hercules, CA, USA). PCR amplification was performed for 40 cycles of 95 °C for 15 s and 60 °C for 60 s. Primers used in the present study are listed in Table 1. Gene-expression levels were normalized to glyceraldehyde 3-phosphate dehydrogenase (*GAPDH*) expression levels.

**Table 1:** Oligonucleotide primers used for real time-polymerase chain reaction

Gene symbol	Sense primer	Antisense primer
*BCL2A1*	5′-GATAAGGCAAAACGGAGGCTG-3′	5′-AGTATTGCTTCAGGAGAGATAGC-3′
*CCL3*	5′-CTCTGCAACCAGTTCTC-3′	5′-AATTCTGTGGAATCTGCC-3′
*CXCL1*	5′-TGCTGAACAGTGACAAATC-3′	5′- CTTCTGTTCCTATAAGGGC-3′
*CXCL2*	5′-GCAGAAAGCTTGTCTCAACCC-3′	5′-CTCCTTCAGGAACAGCCACCAA-3′
*F13A1*	5′-AACAGCCACAACCGTTACACC-3′	5′-TTGGATCAGCACCGCCTCTTT-3′
*F13B*	5′-AAGCGGCTACCTTCTCCATGG-3′	5′-CCAATATCCCGTCTGCAACAG-3′
*GAPDH*	5′-CTTTTGCGTCGCCAG-3′	5′-TTGATGGCAACAATATCCAC-3′
*CXCL8*	5′-GTTTTTGAAGAGGGCTGAG-3′	5′-TTTGCTTGAAGTTTCACTGG-3′
*STAT1*	5′-TGGCAGTCTGGCGGCTGAATT-3′	5′-CAAACCAGGCTGGCACAATT-3′
*TRAF1*	5′-GATGGCACTTTCCTGTGGAAG-3′	5′-ACAGCCGCAGGCACAACTTGT-3′

### Next-generation sequencing (RNA-seq)

RNA sequence library preparation, sequencing, mapping, and gene-expression analyses were performed using DNAFORM (Yokohama, Kanagawa, Japan). The total RNA was quantified using Nanodrop (Thermo Fisher, Waltham, MA, USA), and its quality was assessed using a Bioanalyzer (Agilent Technologies) to ensure that the RNA integrity number (RIN) was above 7.0. Double-stranded cDNA libraries (RNA-seq libraries) were prepared using SMARTer Stranded Total RNA Sample Prep Kit - HI Mammalian (Clontech, Kusatsu, Japan), according to the manufacturer’s instructions. RNA-seq libraries were sequenced using paired-end reads (50nt for read1 and 25nt for read2) on a NextSeq 500 instrument (Illumina, San Diego, CA, USA). The raw reads obtained were trimmed and quality-filtered using Trim Galore! (version 0.4.4), Trimmomatic (version 0.36), and Cutadapt (version 1.16). The trimmed reads were mapped to the human GRCh38 genome using STAR (version 2.7.2b). Reads on the annotated genes were counted using featureCounts (version 1.6.1). Fragments per kilobase of exon per million reads mapped (FPKM) values were calculated from mapped reads by normalizing them to total counts and transcripts. Genes with read counts <10 were excluded and normalized to the housekeeping genes. Relative expression levels were calculated using the following formula: (1) Log_2_[(the gene read count of each sample)/(total average count of the gene)], and then, (2) the difference between the mean value of (1) in F13B IVT mRNA transfected cells and that in EGFP IVT mRNA transfected cells was calculated. The top-regulated genes were determined based on larger fold changes.

### Live cell counting against a cytotoxic agent using MH7A cell line

MH7A cells in 96-well plates were transfected using mock IVT mRNA, F13B IVT mRNA, and Lipofectamine MessengerMAX (Thermo Fisher Scientific), according to the manufacturers’ instructions, as noted above. At 24 h After transfection, hydrogen peroxide (H_2_O_2_) solution (1–10 mM at a final concentration) was added to induce apoptotic cell death. At 3 h after stimulation, Calcein-AM (Cell Couting Kit-F, Dojindo Laboratories, Kumamoto, Japan) was added on the treated cells. According to the manufacturer’s protocol, Calcein fluorescence was measured using a SpectraMax iD3 system (Molecular Devices) at excitation and emission wavelengths of 485 and 525 nm, respectively.

### Statistical analyses

All statistical analyses were conducted using the student’s t-test. All graphs show the results of representative experiments from several individual experiments. The results were analyzed and processed using GraphPad Prism 9 (GraphPad, Inc., La Jolla, CA, USA).

## Results

### FXIII expression in synovial tissue

**Fig. 1:**
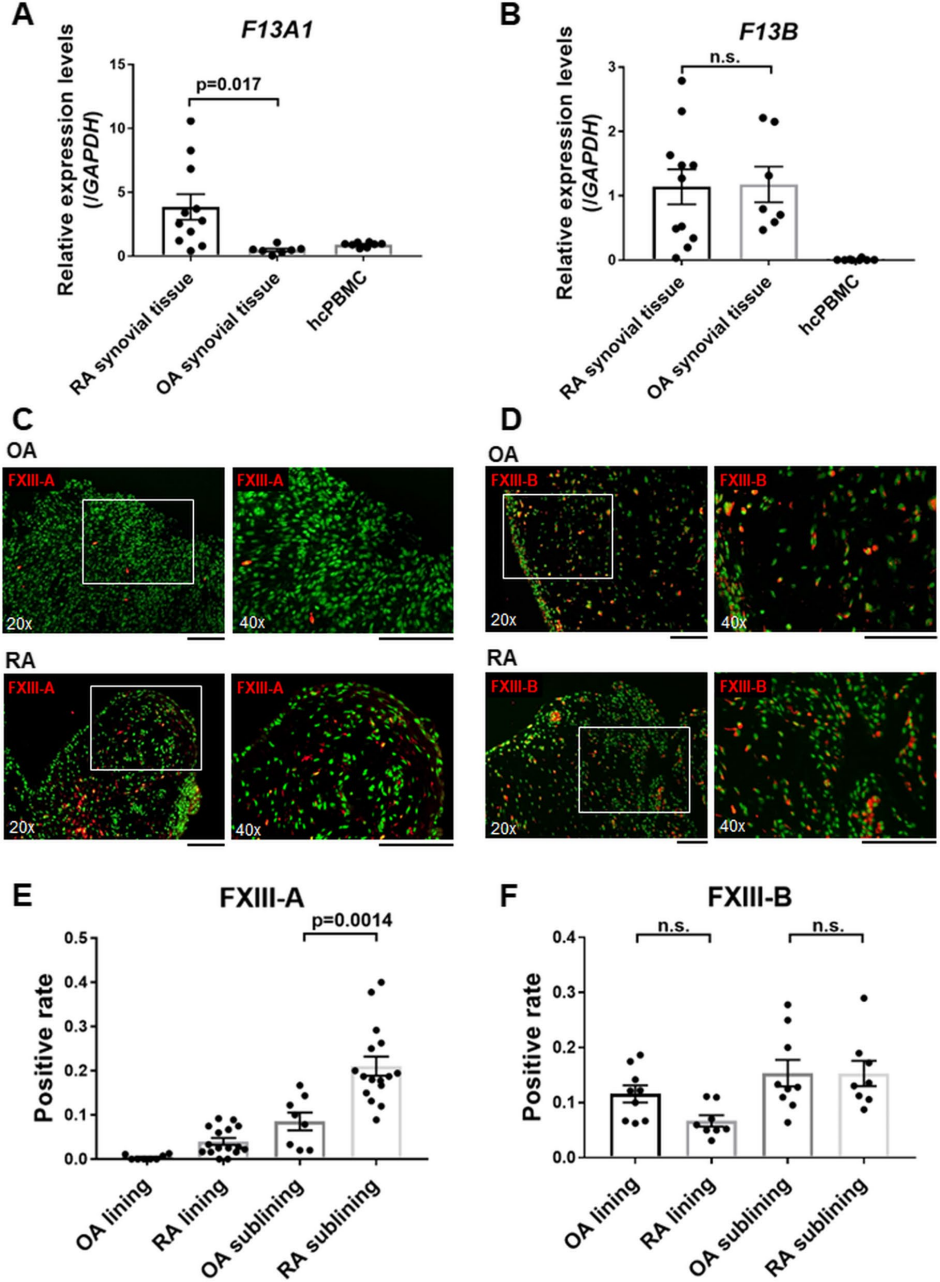
Synovial tissues express FXIII-A and FXIII-B. **A**, **B** Expression levels of **A** *F13A1* and **B** *F13B* in whole synovial tissue collected from patients with rheumatoid arthritis (RA), osteoarthritis (OA), and healthy control peripheral blood mononuclear cells (*n* = 11, 7, and 8, respectively), detected with RT-qPCR. **C**, **D** Fluorescent IHC for synovial tissues collected from patients with OA and RA. Cell nuclei were stained with acridine orange. **C** Anti-FXIII-A antibodies (red) and nuclear staining (green) were merged. **D** Anti-FXIII-B antibodies (red) and nuclear staining (green) were merged. **E** The proportion of FXIII-A-positive cells on the lining and sublining layers in OA (*n* = 8) and RA (*n* = 16) synovial tissues. **F** The proportion of FXIII-B-positive cells on the lining and sublining layers in OA (*n* = 8) and RA (*n* = 8) synovial tissues. **A**, **B**, **E**, **F** Data are presented as mean ± standard error of mean (SEM), *t* test. **C**, **D** Black scale bar = 100 μm

A ProteomicsDB [14] search revealed that FXIII-A was expressed in various tissues throughout the body, primarily in monocytes. In contrast, FXIII-B was expressed only in a limited number of tissues, predominantly in synovial fluid (Suppl. Fig. 1A and B). The protein expression level of FXIII-B in synovial fluid was higher than that in liver tissue (Suppl. Fig. 1B). Therefore, subsequent experiments were performed based on the hypothesis that FXIII-B-expressing cells are present in synovial tissues, which produce synovial fluid. RT-qPCR analysis showed that the expression levels of *F13A1* in human synovial tissues harvested from patients with RA were significantly higher than those in OA synovium, which were non-inflammatory control subjects against RA, or human mononuclear cells from healthy volunteers, which were monocytes and lymphocytes under non-pathological conditions. Furthermore, the expression levels of *F13B* in RA and OA synovium were higher than those in human mononuclear cells (Fig. 1A and B). The results indicated that both FXIII-A and FXIII-B are expressed in human synovial tissues. In addition, FXIII-A is particularly expressed at higher levels in RA synovial tissues than that in OA.

IHC staining was performed to verify the locational distribution of FXIII-A and FXIII-B expression in human synovial tissues. The synovial tissue is mainly composed of FLS and synovial macrophages, which form the lining and sublining layers. Under inflammatory conditions, leukocyte infiltration of the synovium is often detected. FXIII-A-positive cells were predominantly observed in the sublining layer. The number of FXIII-A-positive cells in the sublining layer of RA was significantly higher than that of OA (21.1 % and 9.0 %, respectively; Suppl. Fig. 2A, Fig. 1C and E, Suppl. Fig. 3). FXIII-B-positive cells were found in the lining layer (OA, 11.6 % and RA, 7.1 %) and the sublining layer (OA, 15.4 % and RA, 15.4 %; Suppl. Fig. 2B, Fig. 1D and F, Suppl. Fig. 3). There was no difference in the percentage of FXIII-B-positive cells between RA and OA synovium.

### Distribution of FXIII in synovial tissues and the identification of cells expressing them

**Fig. 2:**
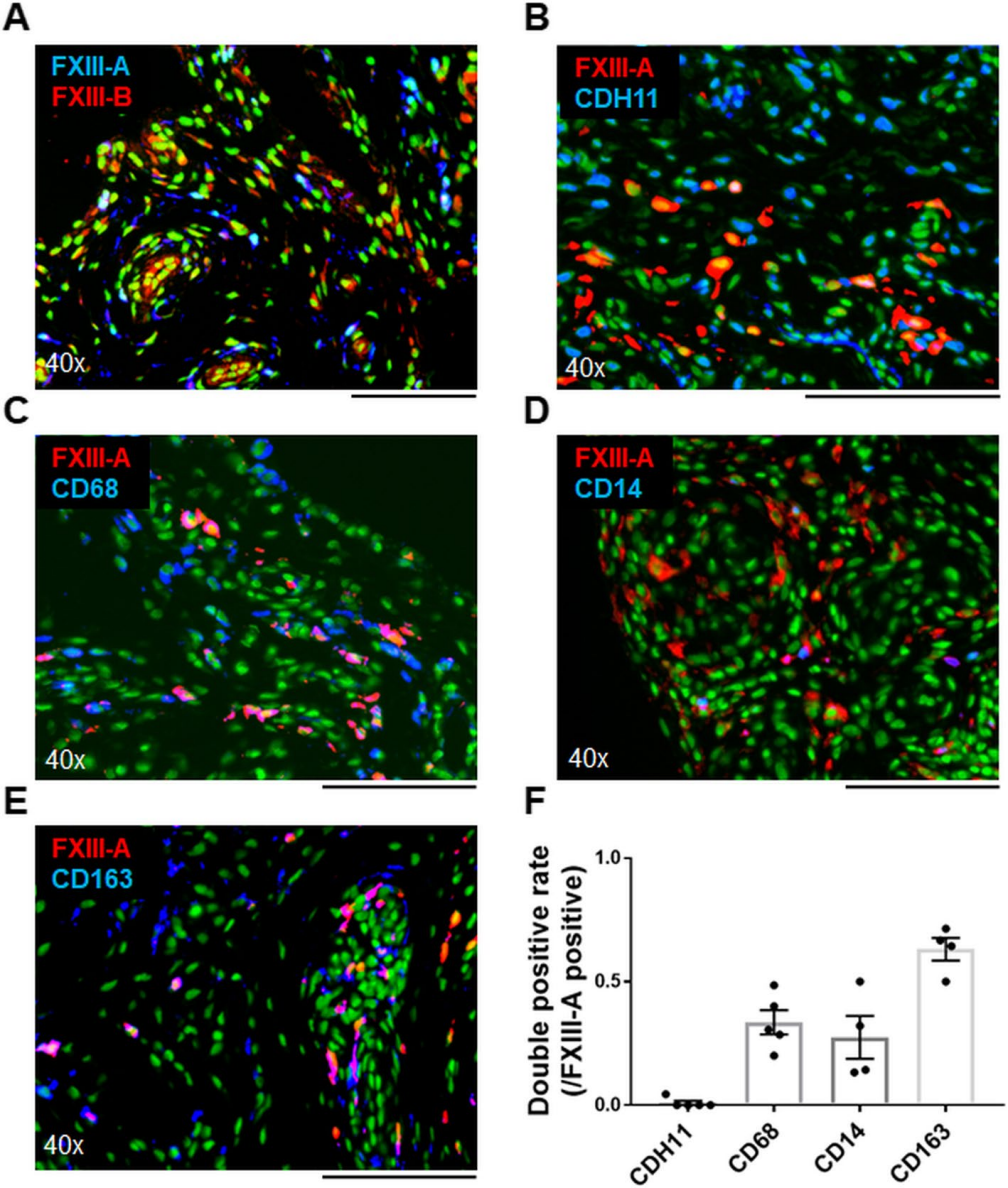
Macrophages produce FXIII-A in RA synovial tissues. **A**–**E** Dual-fluorescent immunohistochemistry (IHC) for synovial tissues collected from patients with RA. Cell nuclei were stained with acridine orange. **A** Merged anti-FXIII-A (blue) and anti-FXIII-B (red) antibodies and nuclear staining (green). **B**–**E** Anti-FXIII-A antibodies (red), nuclear staining (green), and each cell-surface marker antibody (blue) were merged. **B** Anti-CDH11 antibody, **C** anti-CD68 antibody, **D** anti-CD14 antibody, and **E** anti-CD163 antibody. **F** Percentage of positive cells for each antibody among FXIII-A-positive cells (*n* = 4–5, each). Data are presented as mean ± SEM. **A**–**E** Black scale bar = 100 μm

**Fig. 3:**
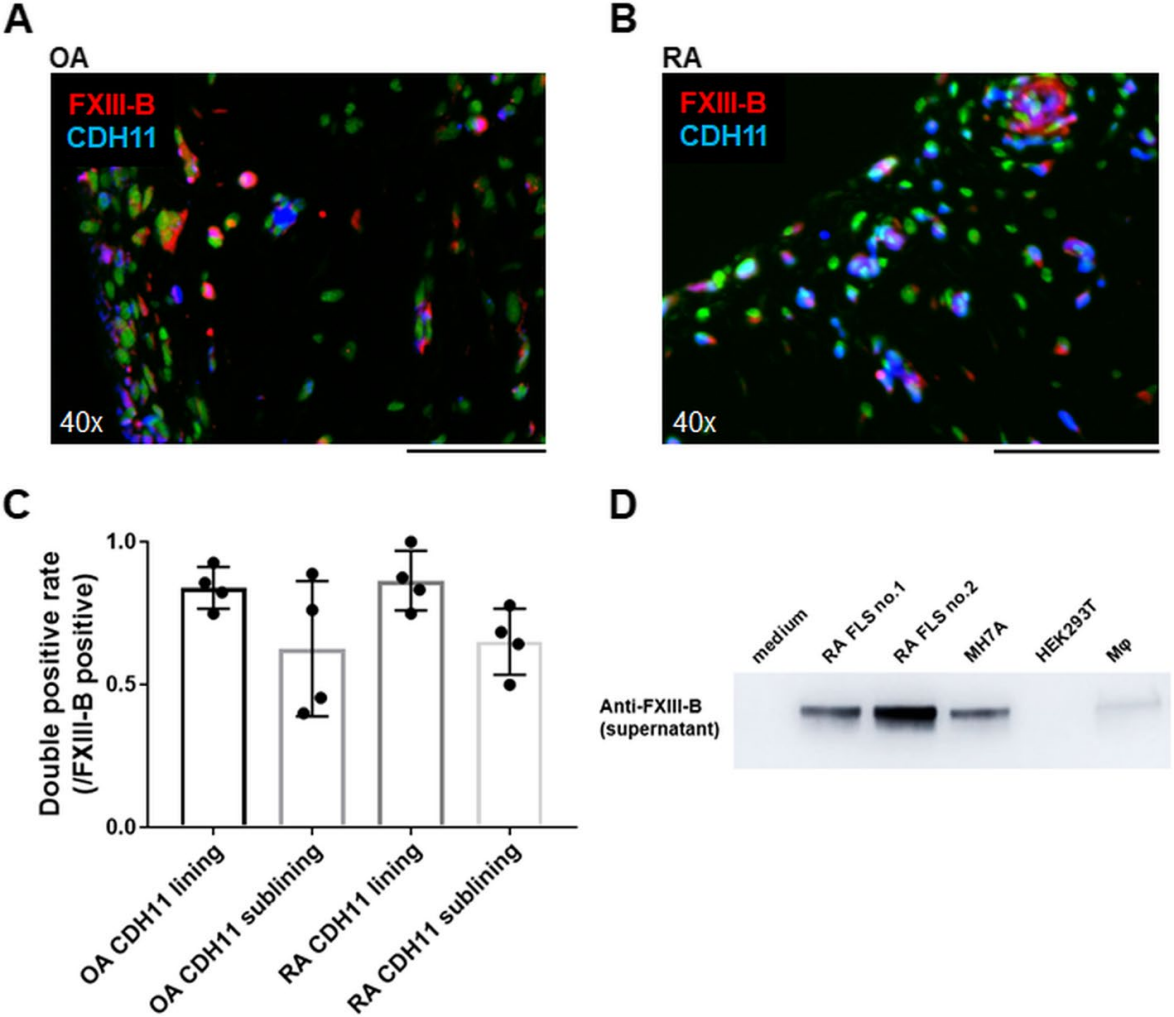
FXIII-B secretion from fibroblast-like synoviocytes. **A**, **B** Merged anti-FXIII-B antibody (red), anti-CDH11 antibody (blue), and nuclear staining (green). Black scale bar = 100 μm. **A** OA (*n* = 4) and **B** RA (*n* = 4). **C** Percentage of anti-CDH11 antibody-positive cells among anti-FXIII-B antibody-positive cells on the lining and sublining layers. Data are presented as mean ± SEM. **D** Analysis of FXIII-B using Western blotting. Culture supernatants obtained from primary cultured FLS (*n*=2, from two patients with RA), MH7A cell line (immortalized rheumatoid fibroblast-like synoviocytes), HEK293T, and monocyte-derived macrophages were collected and concentrated using the acetone method. Western blotting images were cropped to improve the conciseness of the data

Both FXIII-A and FXIII-B are expressed in the synovial tissue. Double-fluorescent IHC staining of RA synovium revealed that only a few cells were double-positive for FXIII-A and FXIII-B, suggesting that they are produced by different cells (Fig. 2A, Suppl. Fig. 4). Double tissue staining was performed using various cell-surface markers. Initially, RA synovial tissues were double-stained with antibodies against FXIII-A and several markers. Staining with FXIII-A and cadherin 11 (CDH11, a marker of FLS) showed that only 0.1 % of cells were double-positive and that FXIII-A-producing cells were not fibroblasts (Fig. 2B, Suppl. Fig. 4). In addition, several lymphocyte markers (CD4, CD8, and CD19) were not merged with FXIII-A at all (Suppl. Fig. 5). While staining for FXIII-A and monocyte- and macrophage-related markers showed a high double-positivity rate of 33.6 % for CD68 and 27.4 % for CD14, and furthermore, staining for CD163 showed the highest double-positivity rate of 63.1 % (Fig. 2C–F, Suppl. Fig. 4). Therefore, monocytes and macrophages, particularly M2 macrophages, are potential candidates for FXIII-A production. Double-staining with FXIII-B and CDH11 for RA and OA synovial tissues revealed that FXIII-B is expressed by the CDH11-positive FLS of both RA and OA (Fig. 3A–C, Suppl. Fig. 6).

### Secretion of FXIII-B from fibroblast-like synoviocytes

The origin of FXIII-B expression was strongly suspected to be synovial fibroblasts based on the proteomics DB and IHC results. To confirm that FLS secretes FXIII-B, we performed a western blot analysis of the culture supernatants. The results showed the presence of abundant FXIII-B protein in culture supernatants of primary cultured FLS and the human synovial fibroblast cell line (MH7A), when compared with other cells such as HEK293T and monocyte-derived macrophages (Fig. 3D). When FLS were cultured in vitro, although the expression declined marginally during passage culture, *F13B* expression was stably detected (Suppl. Fig. 7A). IL-6 stimulation did not affect *F13B* expression levels in FLS (Suppl. Fig. 7B). Therefore, it can be assumed that synovial fibroblasts are one of the sources of FXIII-B production and secretion, and the expression is stable in an inflammatory condition.

### Regulation of FXIII-A expression in monocyte-derived macrophages

**Fig. 4:**
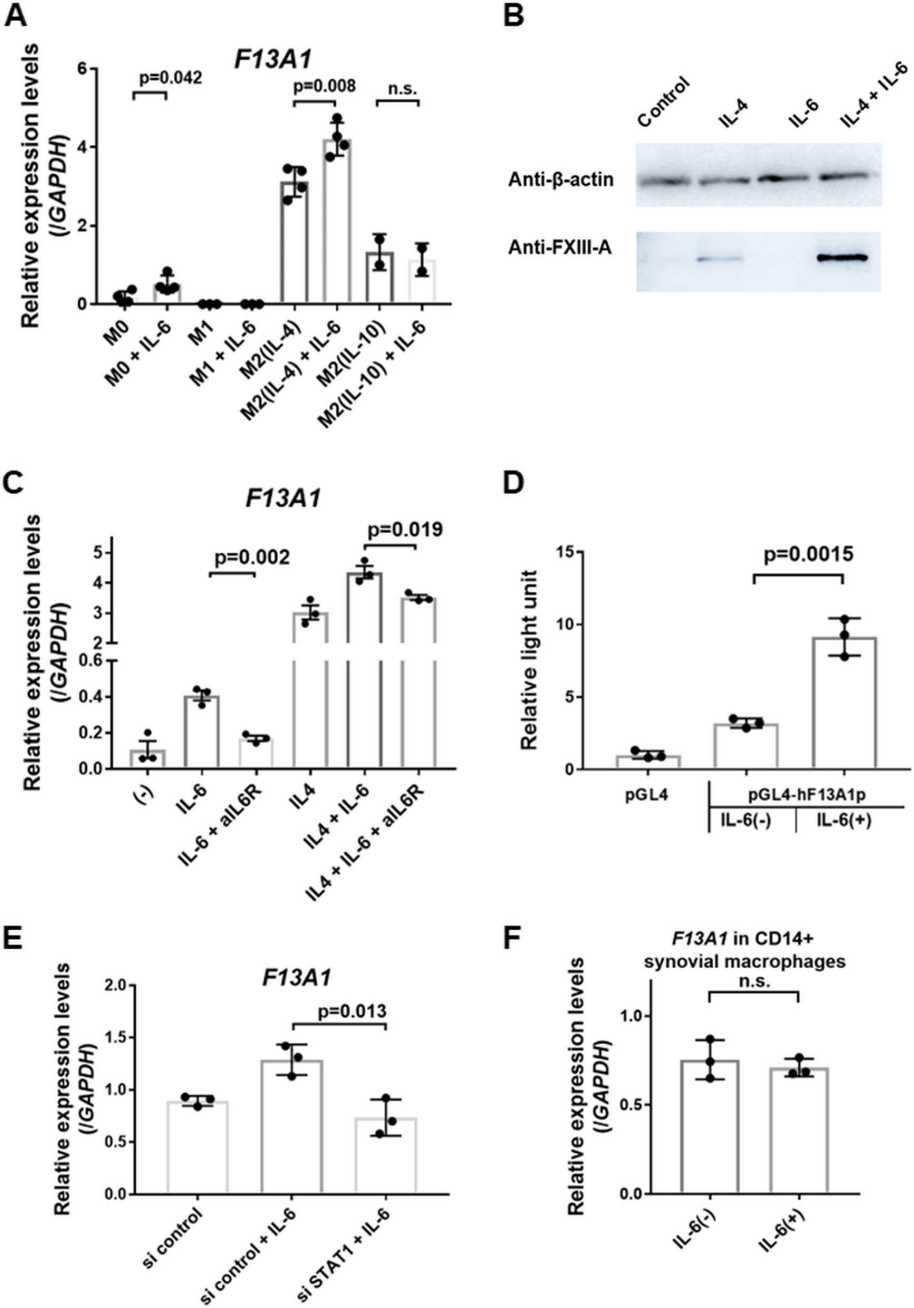
Expression of FXIII-A is upregulated by interleukin-4 and interleukin-6 in monocyte-derived macrophages. **A, C, E, F** *F13A1* expression levels were measured using  RT-qPCR. **A** Cultured monocyte-derived macrophages (M0) were activated [with or without interleukin (IL)-6] with human interferon (IFN)-γ/Lipopolysaccharides (M1), IL-4 [M2(IL-4)], and IL-10 [M2(IL-10)] for another 24 h (*n* = 3). **B** FXIII-A proteins were assayed using Western blotting. Cultured macrophages were stimulated with IL-4, IL-6, and IL-4 + IL-6. Western blotting images were cropped to improve the conciseness of the data. **C** The expression level of *F13A1*. Cultured macrophages were stimulated with or without IL-4, with or without IL-6, and with or without the addition of an anti-IL-6R antibody (*n* = 3). **D** Luciferase assay for *F13A1* promoter region. Reporter plasmids were transfected into HEK293T cells, and then, cells were stimulated by IL-6 + sIL-6Rα. **E** Expression levels of *F13A1*. Cultured macrophages were transfected with small interfering RNA (siRNA) control or siRNA against STAT1 and stimulated with IL-6 (*n* = 3). **F** Expression levels of *F13A1*. CD14-positive synovial cells resembling residual macrophages are stimulated by IL-6 (*n *= 3). **A, B**–**F** Data are presented as mean ± SEM, *t* test

To elucidate the expression of FXIII-A and its regulators, we performed experiments using a macrophage culture system derived from PBMCs. Monocytes harvested from healthy participants were cultured in M-CSF into mature M0 macrophages. Mature macrophages were stimulated with IFN-γ + LPS to differentiate into M1 macrophages, while IL-4 or IL-10 was required to differentiate into M2 macrophages [15] (Suppl. Fig. 8). Our group previously reported a decrease in plasma FXIII-A activity in patients with RA treated with tocilizumab, an anti-IL-6R antibody [6]. We performed a study in which M0 macrophages, M1 macrophages, M2 macrophages (IL-4), and M2 macrophages (IL-10) were treated with IL-6 and evaluated the expression levels of *F13A1*. Both M0 and M2 showed enhanced *F13A1* expression levels in response to IL-6. In particular, the highest upregulation was observed in cells stimulated with IL-4 and IL-6 (Fig. 4A). This phenomenon in IL-4-induced macrophages was also detected using Western blotting (Fig. 4B). Furthermore, *F13A1* expression levels, which were enhanced by IL-6, decreased upon the addition of anti-IL-6R antibody (Fig. 4C). To further confirm the signaling pathway governing IL-6-induced upregulation, we validated luciferase reporter assay for the *F13A1* promoter region (from −783 to + 68 base pairs [bp] relative to the TSS) and measured the expression alteration of *F13A1* using siRNA against STAT1. Luciferase assay showed that IL-6 stimulation upregulated luciferase activity, so that IL-6 could regulate *F13A1* directly (Fig. 4D). In addition, siRNA against STAT1 suppressed IL-6-induced upregulation of *F13A1* expression (Suppl. Fig. 9) (Fig. 4E). The data revealed that FXIII-A is upregulated by IL-6 through STAT1 signaling in monocyte-derived macrophages, particularly in M2 macrophages. We also examined whether FXIII-A expression in CD14-positive synovial cells, to resemble residual macrophages, is affected by IL-6 stimulation, and observed that IL-6 did not upregulate *F13A* levels in the cells (Fig. 4F). Therefore, IL-6-induced FXIII-A expression was detected especifically in monocyte-derived macrophages.

### Elucidation of FXIII-B functions in FLS using IVT mRNA method

**Fig. 5:**
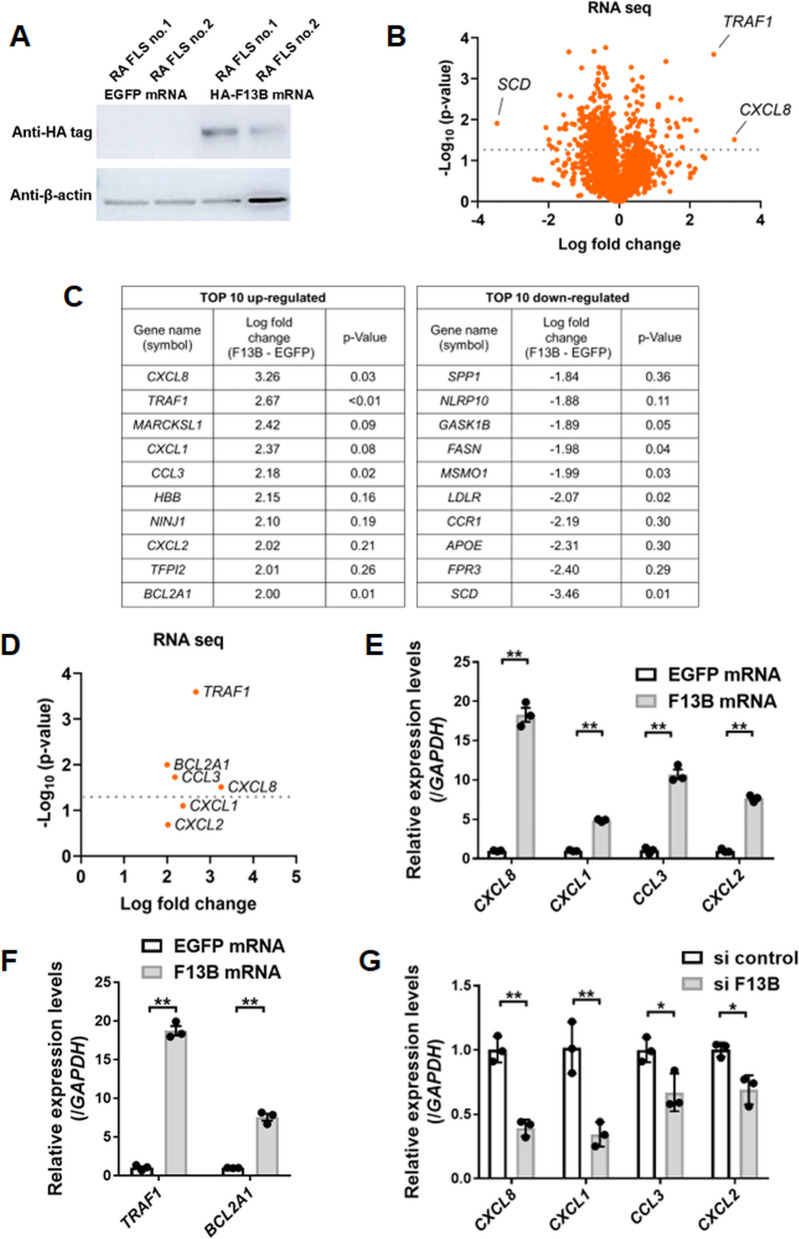
FXIII-B promote chemokine secretion in fibroblast-like synoviocytes. **A** Western blotting. FLS from two patients with RA were transfected with enhanced green fluorescent protein (EGFP) in vitro transcribed (IVT) mRNA or hemagglutinin tagged (HA-tagged) F13B IVT mRNA, then cultured for 24 h. Western blotting images were cropped to improve the conciseness of the data. **B**, **C**, **D** RNA from FLS was collected and analyzed using next-generation sequencing analysis (RNA-seq) (*n *= 2). **B** Volcano plot, **C** Represents the top 10 upregulated or downregulated genes. **D** Volcano plot for chemokine-related and anti-apoptotic-related genes. **E**, **F** RT-qPCR for comparison between FLS transfected with HA-tagged F13B IVT mRNA or those transfected with EGFP IVT mRNA (*n* = 3). **E** Expression levels of *CXCL8*, *CXCL1*, *CCL3*, and *CXCL2.* **F** Expression levels of *TRAF1* and *BCL2A1*. **G** RT-qPCR for comparison between MH7A cells transfected with siRNA against F13B and those transfected with siRNA control (*n* = 3). **E**–**G** Data are presented as mean ± SEM, *t* test, *p<0.05, **p<0.01

FXIII-B has been reported to function as a carrier of FXIII-A [16]. However, its function in synovial tissues has not been reported. To evaluate FXIII-B function, we overexpressed FXIII-B by introducing IVT mRNA into fibroblasts, using a previously reported method [13]. EGFP IVT mRNA was also used as a control. Overexpression was confirmed using Western blotting (Fig. 5A). RNA was collected from these cells and subjected to next-generation sequencing (RNA-seq) (Fig. 5B). The top ten upregulated genes were predominantly chemokine-related (*CXCL8*, *CXCL1*, *CCL3*, and *CXCL2*) and anti-apoptosis-related (*TRAF1* and *BCL2A1*) (Fig. 5C, D). Elevations in these gene expressions were also detected using RT-qPCR (Fig. 5E-F). The siRNA experiments against *F13B* showed that chemokine-related genes were downregulated in parallel with *F13B* knockdown (Fig. 5G). Therefore, FXIII-B can upregulate several chemokine expressions, including *CXCL8*. Moreover, *TRAF1* was upregulated by induction of FXIII-B. TRAF1-knockout dendritic cells reportedly exhibit increased apoptosis and marked deficiency in classical NF-κB activation [17]. Live cell counting in FXIII-B overexpressing MH7A cells showed that FXIII-B inhibited the cell death induced by hydrogen peroxide (H_2_O_2_) (Suppl. Fig. 10). These data indicated that FXIII-B is expressed abundantly in FLS and may contribute to homing of inflammatory cells and synovial proliferation.

## Discussion

The relationship between the disease activity of RA and FXIII has been reported earlier [18–20]. An experiment using a mouse collagen-induced arthritis model revealed that the plasma concentration levels of FXIII are associated with inflammatory arthritis and cartilage and bone destruction [21]. RA-derived FLS, which is characterized by vigorous proliferative capacity, anti-apoptotic effects, IL-6 production, receptor activator of NF-κB ligand (RANKL) expression, and C–X–C motif chemokine (CXCL) production, play significant roles in inflammatory synovial lesions [22, 23]. Upon stimulation with inflammatory cytokines, such as tumor necrosis factor (TNF) and IL-1β, sublining FLS produces a markedly increased level of IL-6 [24]. Bleeding events with FXIII deficiency have been reported in RA patients treated with the anti-IL-6R antibody, tocilizumab [6, 25]. Additionally, our group has previously reported decreased FXIII activity in a group of patients with RA treated with anti-IL-6R antibodies [6]. This response was not observed in those treated with TNF inhibitors. Therefore, we predicted that IL-6, an important mediator of RA, is involved in the regulation of FXIII expression.

In the present study, we aimed to elucidate the mechanism underlying the regulation of FXIII expression by IL-6. Based on the results of a proteomics database, we determined that FXIII is produced in the synovial tissues. IHC staining of synovial tissues showed that FXIII-A and FXIII-B originated from M2 macrophages and FLS, respectively. Furthermore, we elucidated the regulation and function of FXIII subunits, as described below. Therefore, analyzing the mechanism of FXIII expression may contribute to developing new therapeutic strategies for managing side effects associated with anti-IL-6R therapy. Since FXIII deficiency can sometimes prove fatal, elucidating the mechanism of FXIII expression will clarify its pathogenesis and lead to therapeutic development for enhancing the safety of RA treatment.

FXIII-A is expressed in monocytes [8, 9] and macrophages [9]. Monocytes/macrophages can vary phenotypically in response to their microenvironment. The pro-inflammatory M1 phenotype (classical activation) and anti-inflammatory M2 phenotype (alternative activation) are currently recognized [26, 27]. Based on our experimental results, FXIII-A was strongly upregulated in the M2 phenotype, particularly when differentiated by IL-4. This finding is consistent with the results reported by Törocsik et al., wherein IL-4 stimulation upregulates FXIII-A expression in monocyte-derived macrophages [28, 29]. In IL-4-treated M2 macrophages, IL-6 stimulation further upregulated FXIII-A gene expression. IL-6-induced FXIII-A expression was also shown to be upregulated through the STAT1-mediated pathway. Transcription factors involving both IL-4 and IL-6, such as Basic Leucine Zipper ATF-Like Transcription Factor (BATF), in CD4+ lymphocytes have been reported [30]. Further investigation of the regulatory pathway of FXIII-A in monocyte-derived macrophages is required.

It has been reported that FXIII-B is synthesized and secreted by human hepatoma cell lines [11, 31]. In contrast, some authors believed that FXIII-B-expressing cells have been detected in vascular endothelial cells and fibroblasts but not in hepatocytes [12]. In the present study, we performed the elucidation of FXIII-B expression according to a proteomics data base. Fibroblasts secreted FXIII-B in the synovium, which was detected by western blotting of the culture supernatants. To the best of our knowledge, the present study is the first to show FXIII-B expression outside of hepatocytes. Furthermore, although FXIII-B is known to function as a transporter, its other functions remain unclear. In the present study, FXIII-B functions were verified by transducing F13B IVT mRNA in FLS. Our results indicate that FXIII-B in FLS may be involved in an anti-apoptotic effect, which leads to cell proliferation, and the transfer of inflammatory cells induced by chemokines. Sushi domain containing proteins, such as Sushi Domain Containing 2 (SUSD2), have multiple functions [32]. FXIII-B also have 10 sushi domains [33]. Sushi domains of FXIII-B may have chemokine production and anti-apoptotic gene expression functions.

Integration of previously reported FXIII-A functions and our results on FXIII-B functions implied a pathophysiological relationship between RA pathogenesis and FXIII. It has been reported that migration of monocytes from healthy donors’ skin and lung fibroblasts was promoted by extracellular FXIII-A [34]. In our analyzed RNA-seq data, CXCL8 was largely elevated with FXIII-B overexpression. CXCL8 can recruit neutrophils [35]. FXIII-B also contributes to anti-apoptotic function in parallel with TRAF1 expression. Therefore, extracellular FXIII-A and FXIII-B-expressing FLS potentially promote monocyte migration, neutrophil infiltration, and fibroblast proliferation to establish inflammatory joint diseases. In both FXIII-A- and FXIII-B-expressing RA synovium, neutrophils, lymphocytes, and capillary are abundant, when compared with OA synovium, which includes FXIII-B expression without FXIII-A. We presume that FXIII-B alone may be inadequate for joint inflammation development, whereas other factors, excluding FXIII-B expression, such as immune complex deposition, aggressive leukocyte infiltration, vigorous angiogenesis, and extracellular FXIII-A, could contribute to the establishment of RA synovial lesion.

A limitation of the present study was that we could not determine the expression pattern of FXIII-A in non-inflammatory or healthy conditions other than RA. Further investigation in this regard is required in the future.

## Conclusions

Our results showed that FXIII-A was expressed in macrophages, and FXIII-B was stably expressed in synovial tissues and secreted from FLS. FXIII-A expression was upregulated by IL-4 and IL-6. Moreover, the constant expression of FXIII-B in FLS may inhibit apoptosis and result in the secretion of several chemokines.

## Supplementary Information

**Additional file 1: Supple. Fig. 1A.** FXIII-A expression on ProteomicsDB. FXIII-A was secreted from mononuclear cells and platelets. References from ProteomicsDB (https://www.ProteomicsDB.org), accessed April/13/2020. **Supple. Fig. 1B.** FXIII-B expression on ProteomicsDB. FXIII-B was mostly distributed in the synovial fluid. References from ProteomicsDB (https://www.ProteomicsDB.org), accessed April/13/2020. **Supple. Fig. 2A.** Immunohistochemical staining for FXIII-A. Immunohistochemistry (IHC) staining of synovial tissues from patients with OA and RA. Synovial tissues were treated with anti-FXIII-A antibody (red). Magnification of objective lens: 10×. Black scale bar = 100 μm. **Supple. Fig.2B.** Immunohistochemical staining for FXIII-B. Immunohistochemistry (IHC) staining of synovial tissues from patients with OA and RA. Synovial tissues were treated with anti-FXIII-B antibody (red). Magnification of objective lens: 10×. Black scale bar = 100 μm. **Supple. Fig. 3.** Hematoxylin and eosin staining for synovial tissues. Hematoxylin and eosin staining in patients with OA and RA synovial tissues. Magnification of objective lens 10×. Black scale bar = 100 μm. **Supple. Fig. 4.** Dual-fluorescent IHC for RA synovial tissues. Supplementary images for fluorescence staining in Fig.2. IHC was performed using nuclear staining (AO, acridine orange) and each antibody. Black scale bar = 100 μm. **Supple. Fig. 5.** Fluorescent IHC staining for FXIII-A & CD4/CD8/CD19. IHC images in synovium of RA treated with anti-FXIII-A antibody (blue), nuclear staining (green; AO, acridine orange) and (A) anti-CD4 (red) or (B) anti-CD8 (red) or (C) anti-CD19 (red) antibodies. Black scale bar = 100 μm. **Supple. Fig. 6.** Fluorescent IHC staining for FXIII-B & CDH11. Supplementary images for Fig. 3. Images of fluorescence staining with each antibody and low power field images. Black scale bar = 100 μm. **Supple. Fig. 7A.** Alteration of FXIII-B expression levels in passage culture of FLS. The expression levels of *F13B* in synovial fibroblasts were measured using RT-qPCR. (*n* = 3; mean ± SEM). **Supple. Fig. 7B.** *F13B* expression in FLS is not altered by IL-6 stimulation. The expression levels of *F13B* in synovial fibroblasts with or without IL-6 and sIL-6Rα were measured using RT-qPCR. (*n*=3; mean ± SEM). **Supple. Fig. 8.** The schema of macrophage generation. The schema of monocyte-derived macrophage generation created using BioRender.com. **Supple. Fig. 9.** Knockdown of STAT1 using siRNA. The expression level of *STAT1* was measured using RT-qPCR. Cultured macrophages were transfected with siRNA control or siRNA against *STAT1*, and then, stimulated with human recombinant IL-6 (*n* = 3; mean ± SEM; t-test). **Supple. Fig. 10.** Anti-apoptotic function of FXIII-B overexpression. Live cell counting in FXIII-B-overexpressing MH7A with hydrogen peroxide (H_2_O_2_) stimulation at a final concentration of 1–10 mM. MH7A cells were transfected with mock IVT mRNA or F13B IVT mRNA. (n=3; mean ± SEM; *t-test for mock vs F13B, *p*<0.05).

## Data Availability

The datasets used and/or analyzed during the current study are available from the corresponding author on reasonable request. The RNA-seq raw data are available in the NCBI Sequence Read Archive (SRA) with accession number PRJNA841780.

## Abbreviations

BATF: Basic leucine zipper ATF-like transcription factor
BCL2A1: B cell lymphoma 2-related protein A1
CCL: C–C motif chemokine ligand
CDH11: Cadherin11
CXCL: C–X–C motif chemokine ligand
DMEM: Dulbecco’s modified Eagle’s medium
EGFP: Enhanced green fluorescent protein
FBS: Fetal bovine serum
FLS: Fibroblast-like synoviocytes
FRKM: Fragments per kilobase of exon per million reads mapped
FXIII: Blood coagulation factor XIII
FXIII-A: Factor XIII subunits A
FXIII-B: Factor XIII subunits B
GAPDH: Glyceraldehyde 3-phosphate dehydrogenase
IFN: Interferon
IgG: Immunoglobulin G
IHC: Immunohistochemistry
IL: Interleukin
IL-6R: Interleukin-6 receptor
IVT: In vitro transcribed
LPS: Lipopolysaccharides
M-CSF: Macrophage colony-stimulating factor
OA: Osteoarthritis
PBMCs: Peripheral blood mononuclear cells
RA: Rheumatoid arthritis
RANKL: Receptor activator of NF-κB ligand
RIN: RNA integrity number
RPMI 1640: Roswell Park Memorial Institute 1640
RT-qPCR: Reverse transcription-quantitative polymerase chain reaction
siRNA: Small interfering RNA
STAT1: Signal transducers and activator of transcription 1
TNF: Tumor necrosis factor
TRAF1: TNF receptor associated factor 1
TSS: Transcription start site
