# Supplementary material for: Expression of factor XIII originating from synovial fibroblasts and macrophages induced by interleukin-6 signaling
Source: Inflamm Regen. 2023 Jan 6;43:2. doi: 10.1186/s41232-022-00252-4 (PMC9817275; doi:10.1186/s41232-022-00252-4)

Supplementary Figures

Expression of factor XIII originating from synovial fibroblasts and macrophages induced by interleukin-6 signaling

Watanabe H and Mokuda S, et al.

- Supplementary Figure 1A, 1B, 2A, 2B, 3, 4, 5, 6, 7A, 7B, 8, 9, 10

- Uncropped blotting data

Supplementary Figure 1A
FXIII-A expression on ProteomicsDB.

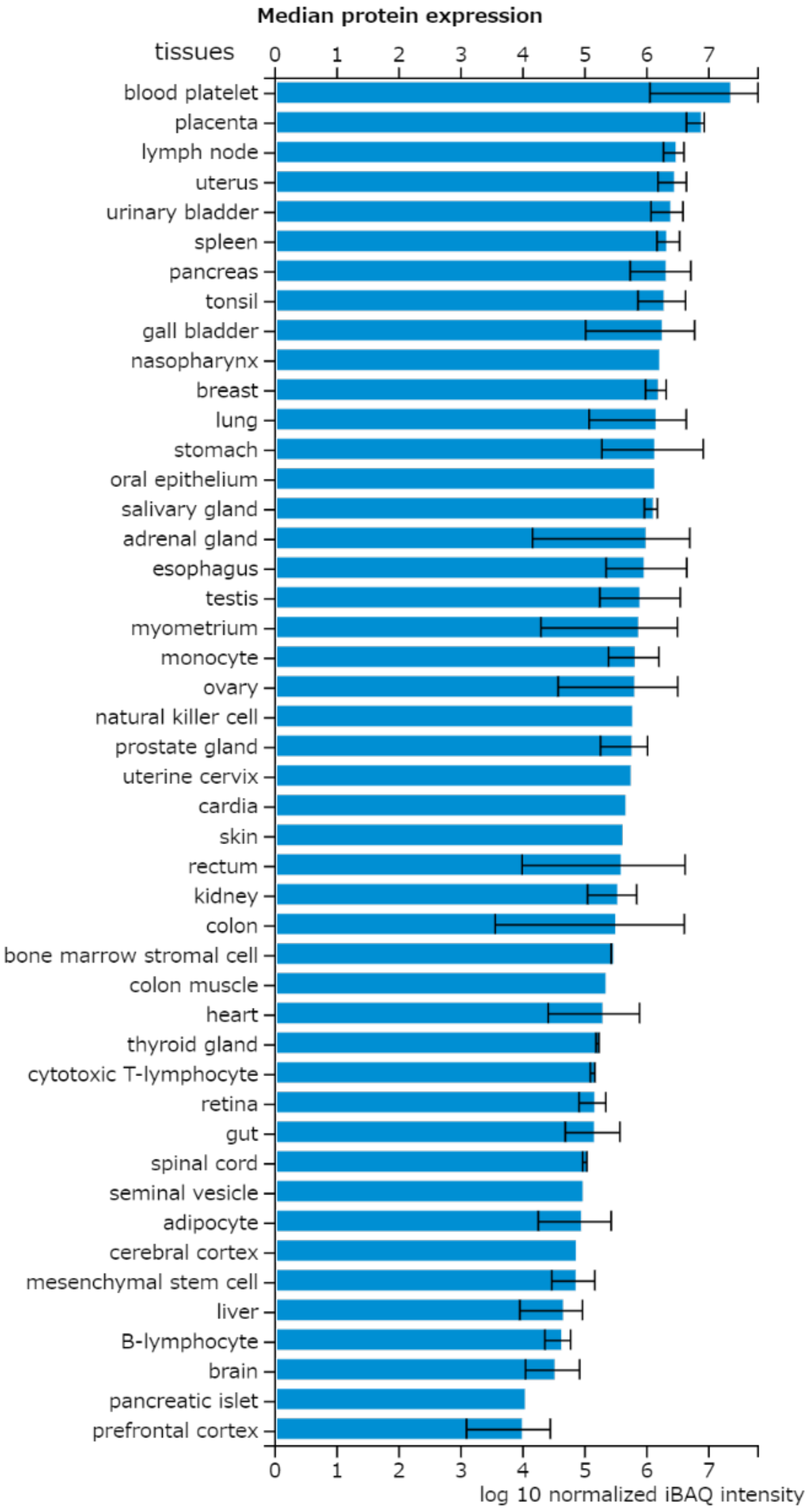

FXIII-A was secreted from mononuclear cells and platelets. References from ProteomicsDB (https://www.ProteomicsDB.org), accessed April/13/2020.

Supplementary Figure 1B
FXIII-B expression on ProteomicsDB.

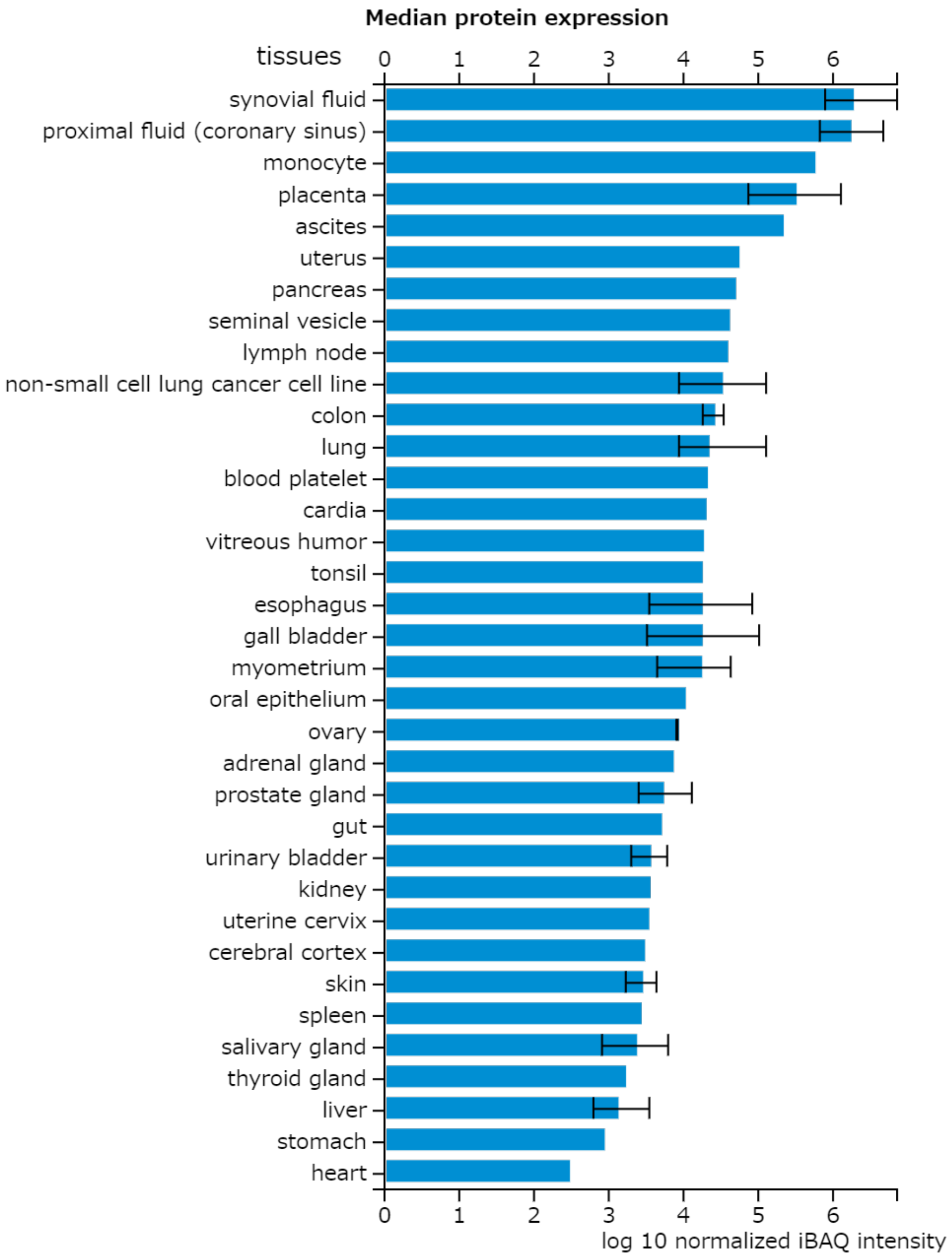

FXIII-B was mostly distributed in the synovial fluid.
References from ProteomicsDB (https://www.ProteomicsDB.org), accessed April/13/2020.

Supplementary Figure 2A
Immunohistochemical staining for FXIII-A

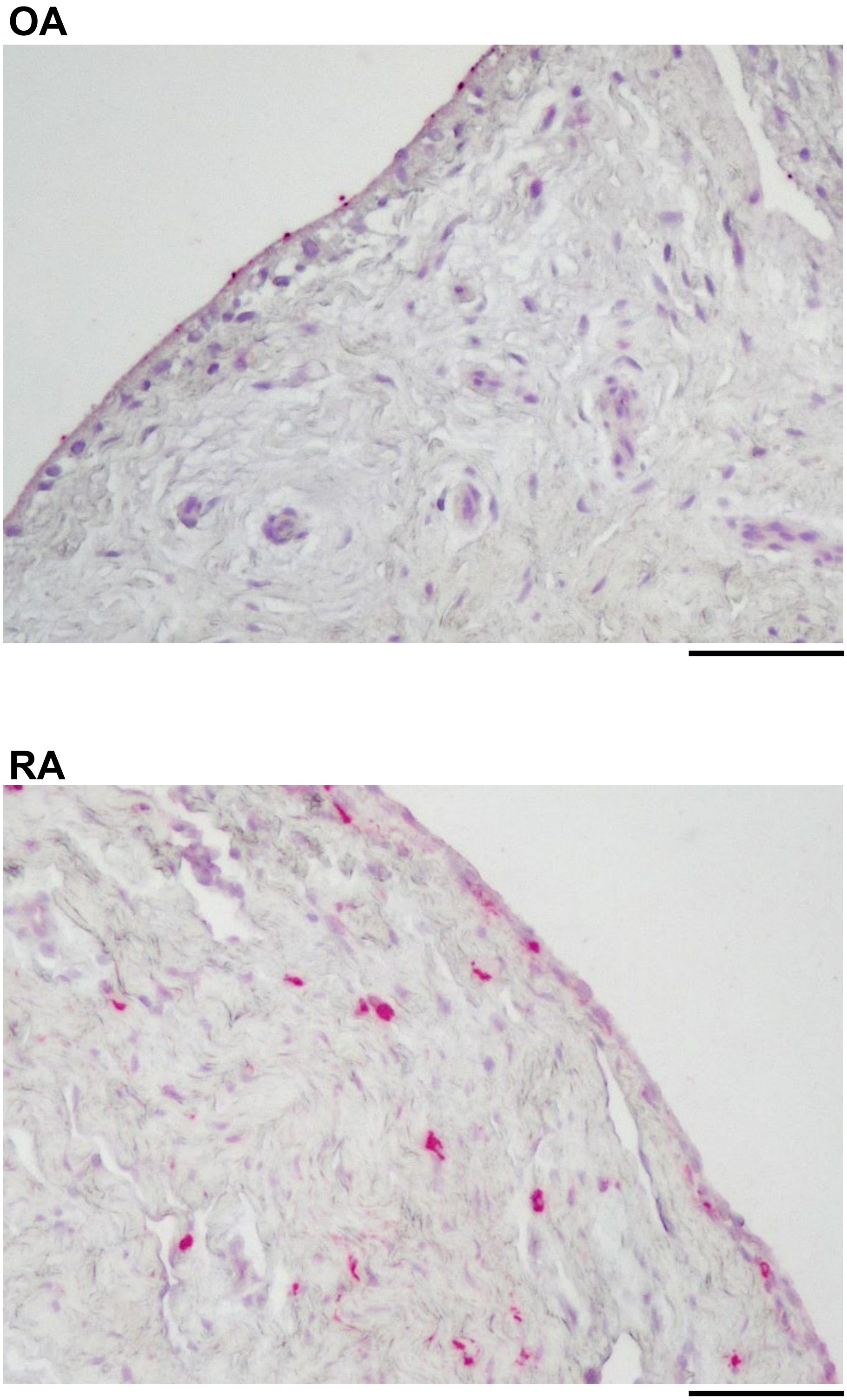

Immunohistochemistry (IHC) of synovial tissues from patients with OA and RA. Synovial tissues were treated with anti-FXIII-A antibody (red). Magnification of objective lens: 10×. Black scale bar = 100 µm.

Supplementary Figure 2B
Immunohistochemical staining for FXIII-B

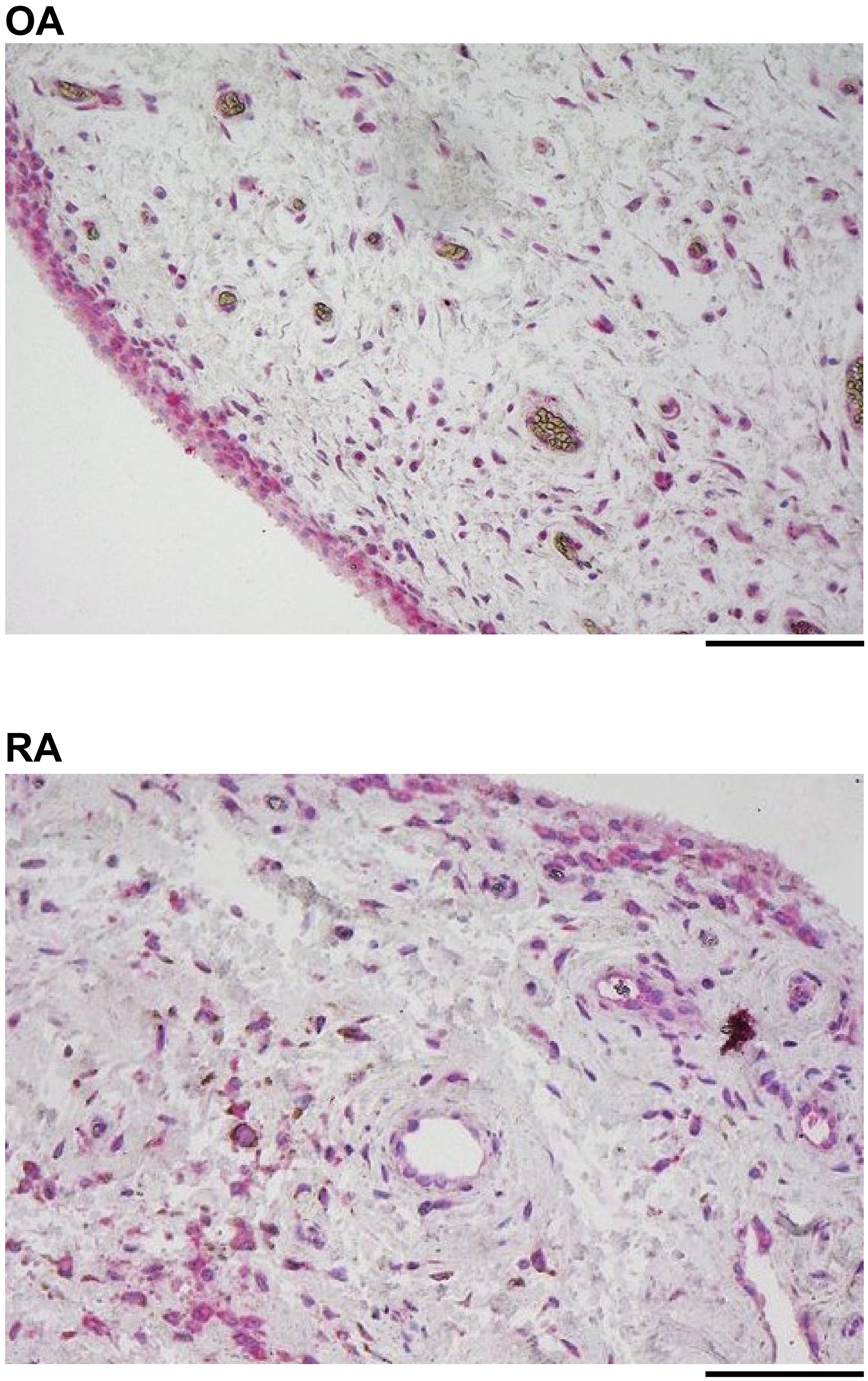

Immunohistochemistry (IHC) of synovial tissues from patients with OA and RA. Synovial tissues were treated with anti-FXIII-B antibody (red). Magnification of objective lens: 10×. Black scale bar = 100 μm.

Supplementary Figure 3
Hematoxylin and eosin staining for synovial tissues

For Fig. 1C
(OA)

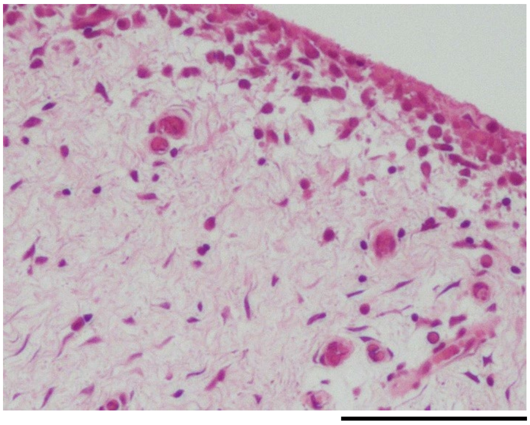

For Fig. 1D
(OA)

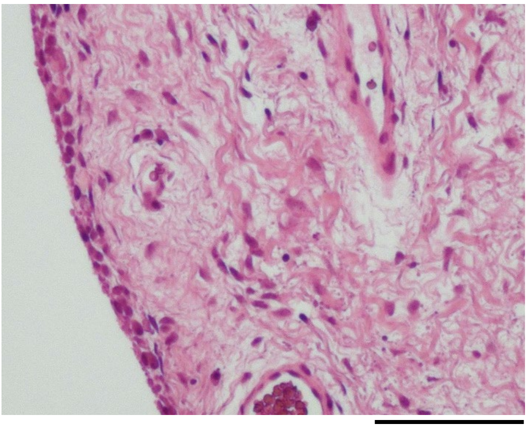

For Fig. 1C
(RA)

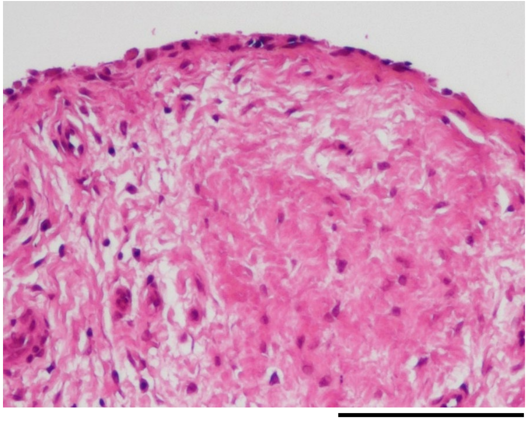

For Fig. 1D
(RA)

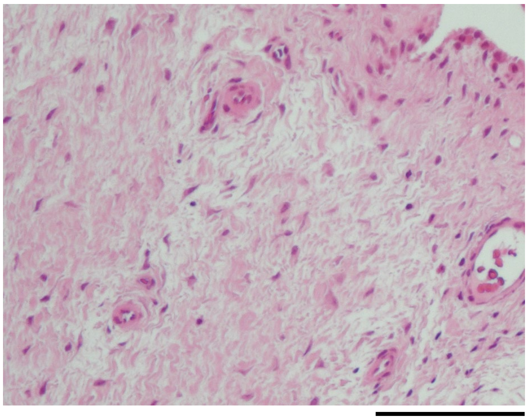

Hematoxylin and eosin staining in patients with OA and RA synovial tissues. Magnification of objective lens: 10×. Black scale bar = 100 μm.

Supplementary Figure 4
Dual-fluorescent IHC for RA synovial tissues

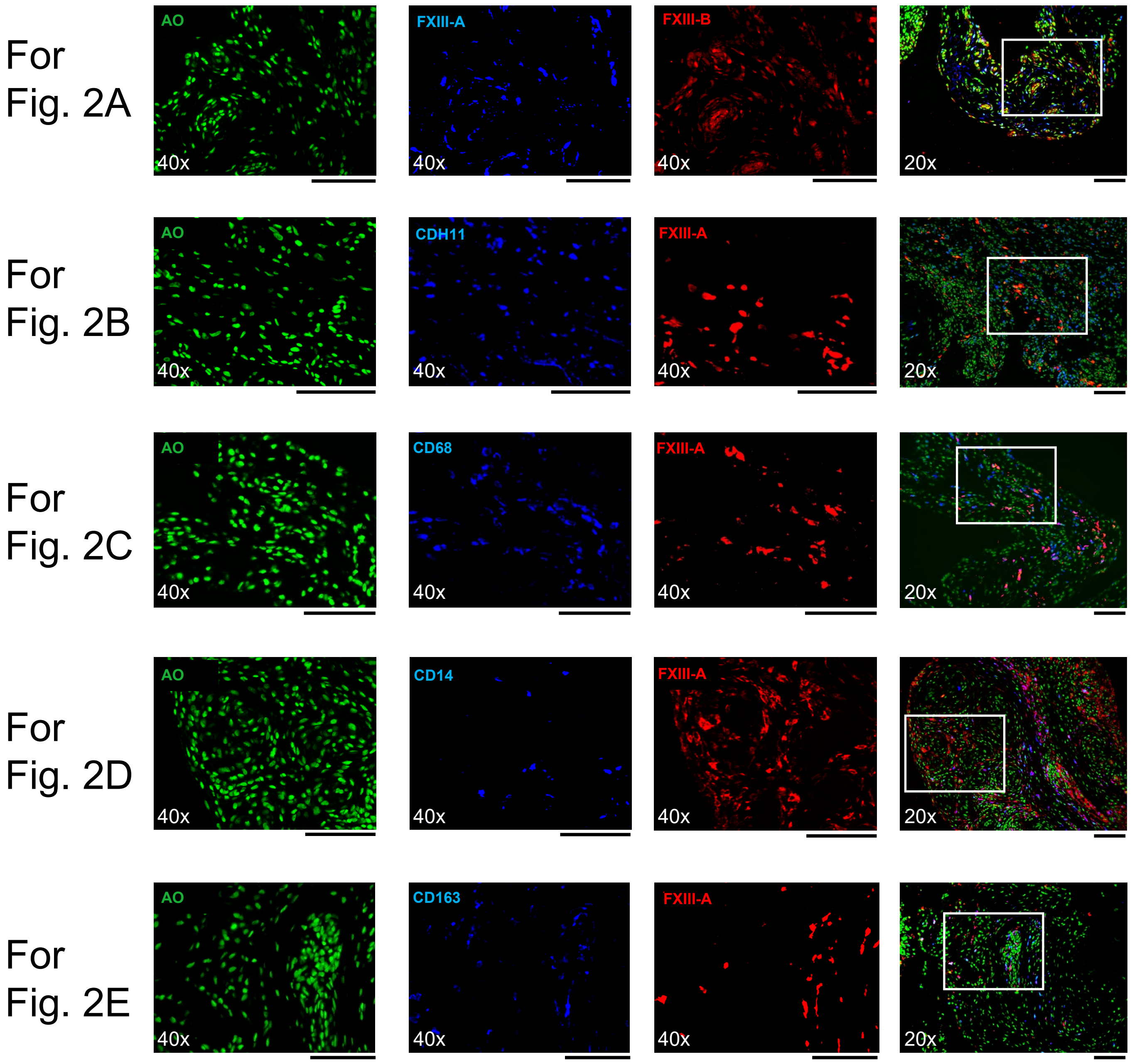

Supplementary images for fluorescence staining in Fig.2. IHC was performed using nuclear staining (AO, acridine orange) and each antibody. Black scale bar = 100 μm.

Supplementary Figure 5
Fluorescent IHC staining for FXIII-A & CD4/CD8/CD19

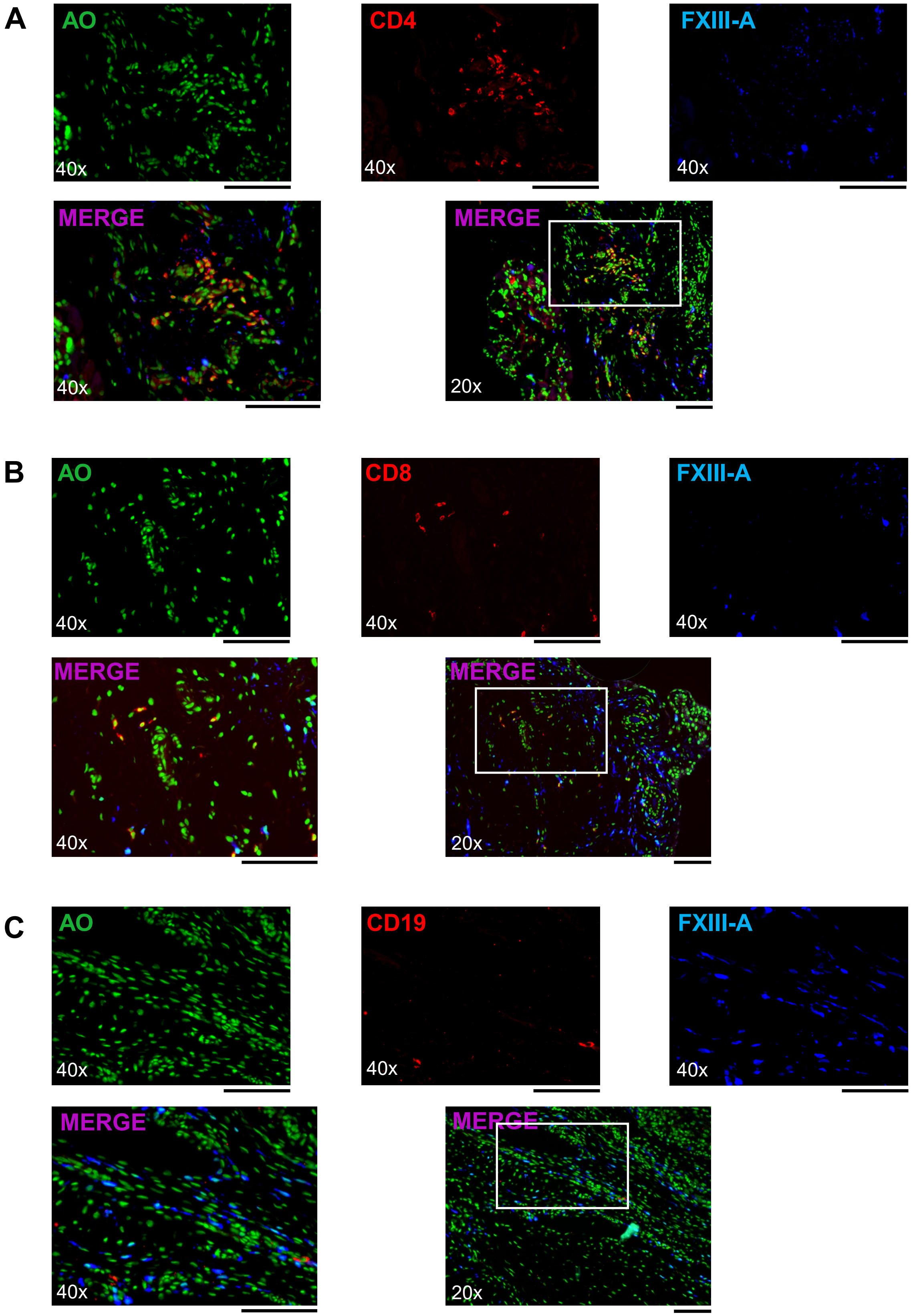

IHC images in synovium of RA treated with anti-FXIII-A antibody (blue), nuclear staining (green; AO, acridine orange) and (A) anti-CD4 (red) or (B) anti-CD8 (red) or (C) anti-CD19 (red) antibodies. Black scale bar = 100 μm.

Supplementary Figure 6
Fluorescent IHC staining for FXIII-B & CDH11

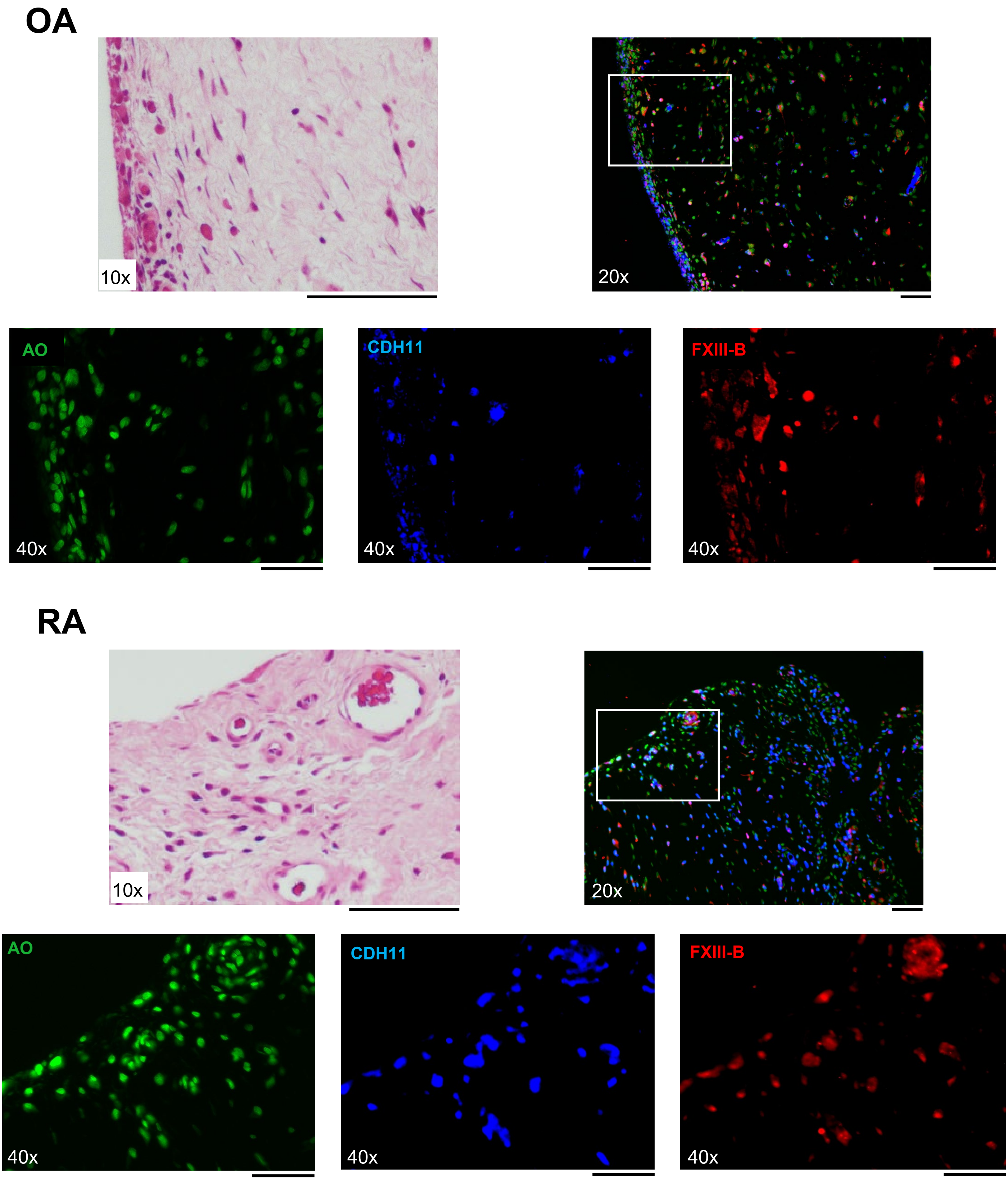

Supplementary images for Fig. 3. Images of fluorescence staining with each antibody and low power field images. Black scale bar = 100 μm.

Supplementary Figure 7A
Alteration of *F13B* expression levels in passage culture of FLS

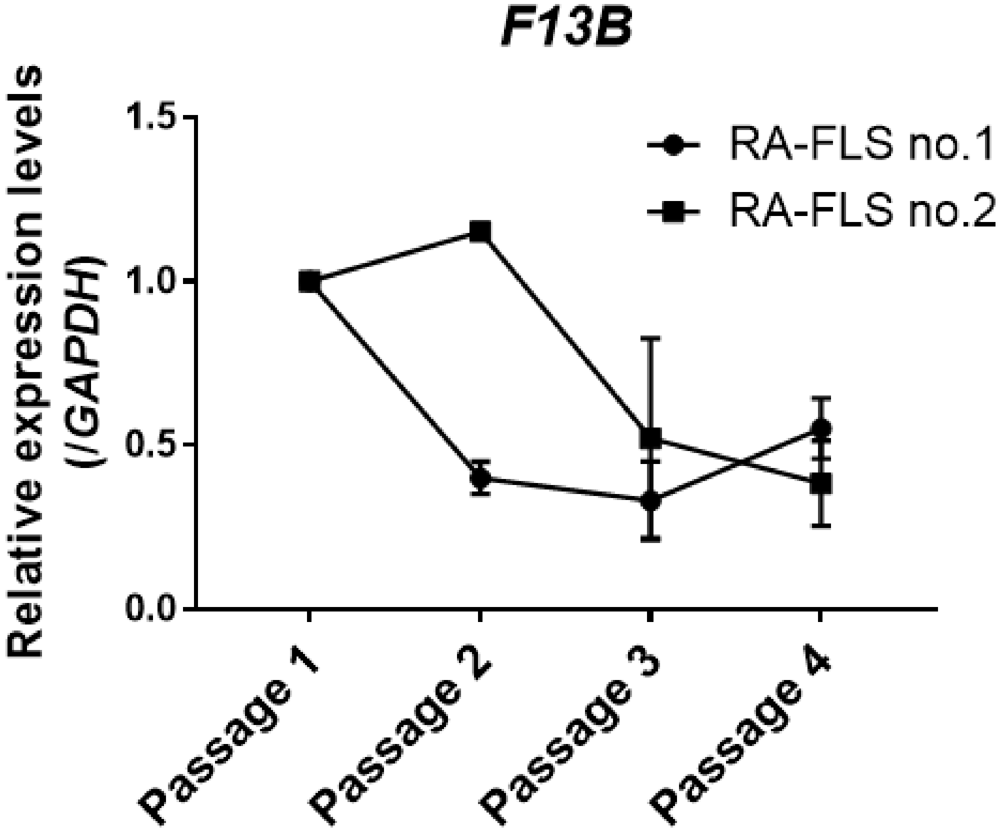

The expression levels of *F13B* in synovial fibroblasts were measured using RT-qPCR. (n=3; mean ± SEM).

Supplementary Figure 7B
***F13B* expression in FLS is not altered by IL-6 stimulation**

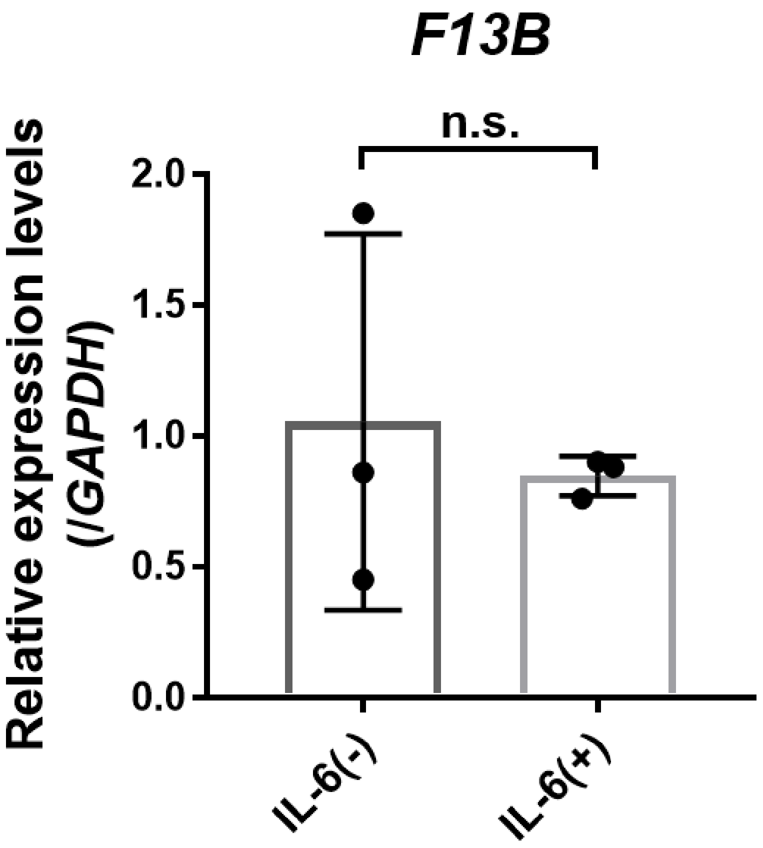

The expression levels of *F13B* in synovial fibroblasts with or without IL-6 and sIL-6Rα were measured using RT-qPCR. (n=3; mean ± SEM).

Supplementary Figure 8
The schema of macrophage generation

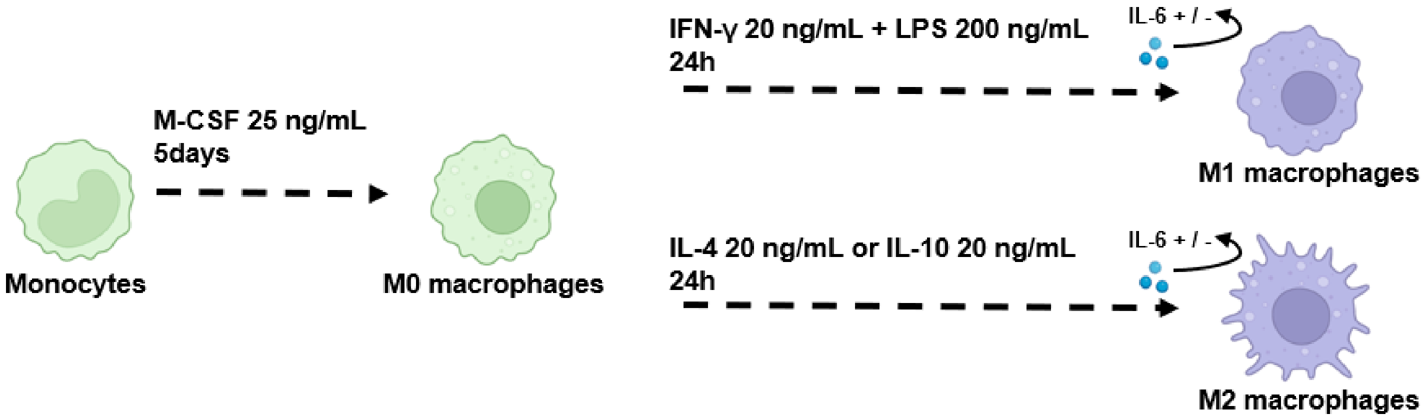

The schema of monocyte-derived macrophage generation created using BioRender.com.

Supplementary Figure 9
Knockdown of STAT1 using siRNA

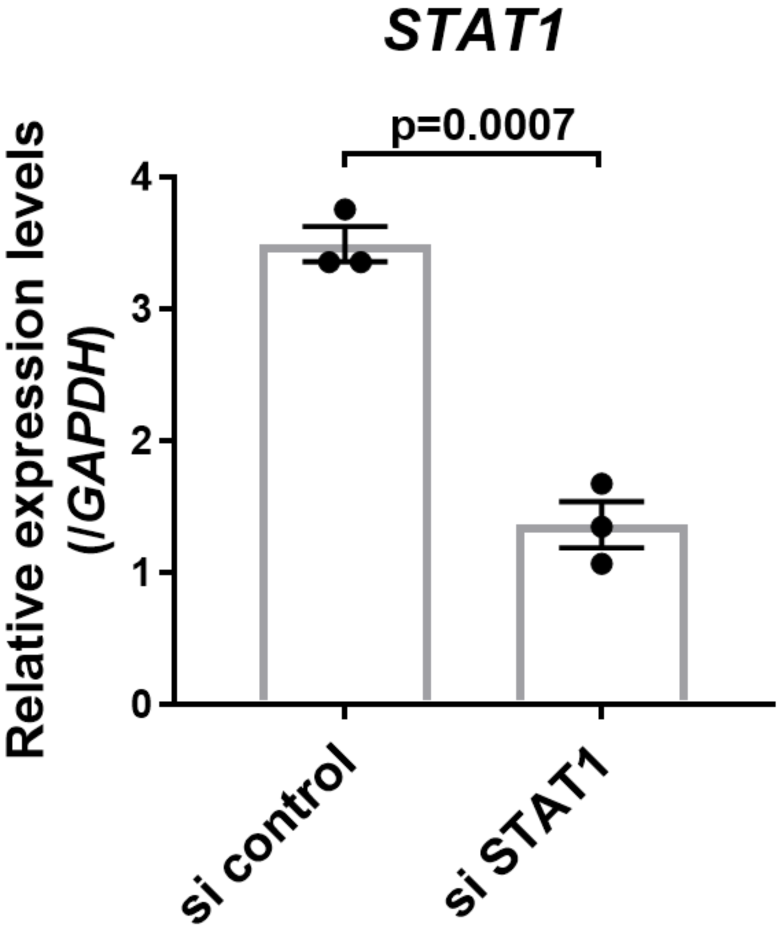

The expression level of *STAT1* was measured using RT-qPCR. Cultured macrophages were transfected with siRNA control or siRNA against *STAT1*, and then, stimulated with human recombinant IL-6 (n = 3; mean ± SEM; t-test).

Supplementary Figure 10
Anti-apoptotic function of FXIII-B overexpression

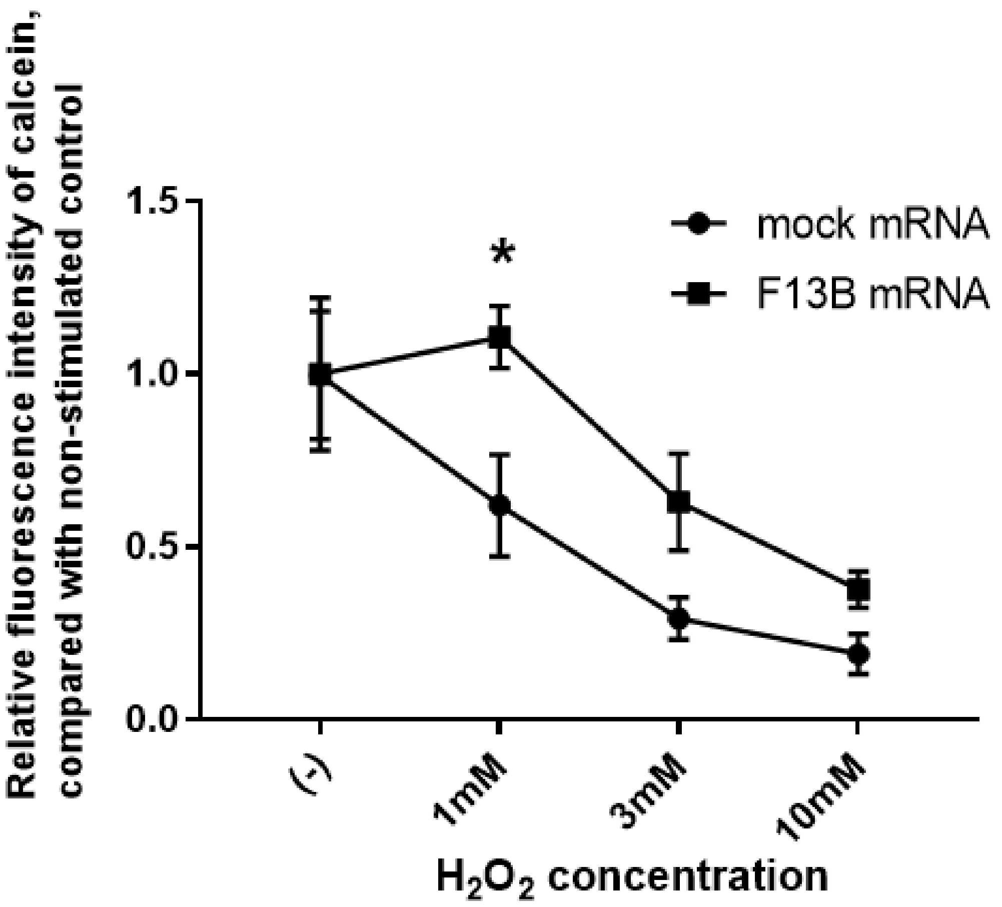

Live cell counting in FXIII-B-overexpressing MH7A with hydrogen peroxide (H_2O_2) stimulation at a final concentration of 1–10 mM. MH7A cells were transfected with mock IVT mRNA or F13B IVT mRNA. ($n=3$; mean ± SEM; *t-test for mock vs F13B, $p<0.05$).

Supplementary images (Uncropped blotting data)

Anti-FXIIIB

Fig. 3D

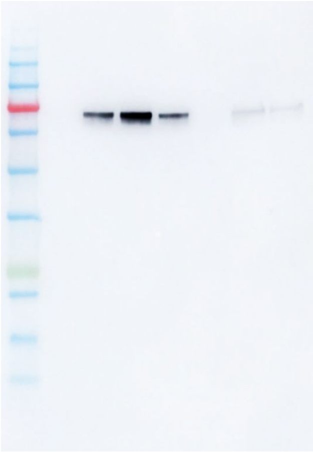

Anti-β-actin

Anti-F13A

Fig. 4B

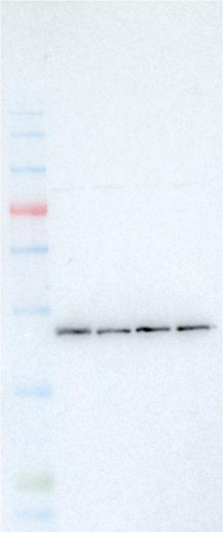

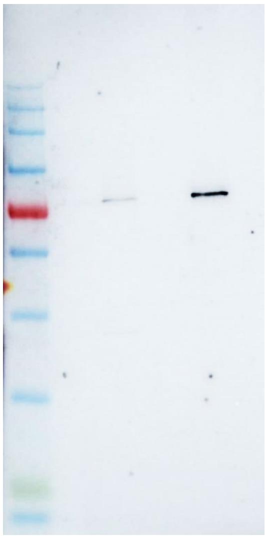

Anti-HA tag

Anti-β-actin

Fig. 5A

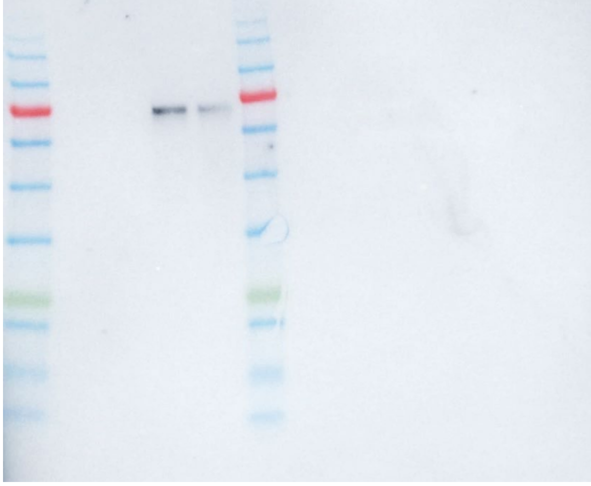

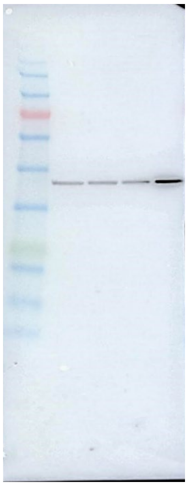

Supplement: Supplementary file 1 — Additional file 1: Supple. Fig. 1A. FXIII-A expression on ProteomicsDB. FXIII-A was secreted from mononuclear cells and platelets. References from ProteomicsDB (https://www.ProteomicsDB.org), accessed April/13/2020. Supple. Fig. 1B. FXIII-B expression on ProteomicsDB. FXIII-B was mostly distributed in the synovial fluid. References from ProteomicsDB (https://www.ProteomicsDB.org), accessed April/13/2020. Supple. Fig. 2A. Immunohistochemical staining for FXIII-A. Immunohistochemistry (IHC) staining of synovial tissues from patients with OA and RA. Synovial tissues were treated with anti-FXIII-A antibody (red). Magnification of objective lens: 10×. Black scale bar = 100 μm. Supple. Fig.2B. Immunohistochemical staining for FXIII-B. Immunohistochemistry (IHC) staining of synovial tissues from patients with OA and RA. Synovial tissues were treated with anti-FXIII-B antibody (red). Magnification of objective lens: 10×. Black scale bar = 100 μm. Supple. Fig. 3. Hematoxylin and eosin staining for synovial tissues. Hematoxylin and eosin staining in patients with OA and RA synovial tissues. Magnification of objective lens 10×. Black scale bar = 100 μm. Supple. Fig. 4. Dual-fluorescent IHC for RA synovial tissues. Supplementary images for fluorescence staining in Fig.2. IHC was performed using nuclear staining (AO, acridine orange) and each antibody. Black scale bar = 100 μm. Supple. Fig. 5. Fluorescent IHC staining for FXIII-A & CD4/CD8/CD19. IHC images in synovium of RA treated with anti-FXIII-A antibody (blue), nuclear staining (green; AO, acridine orange) and (A) anti-CD4 (red) or (B) anti-CD8 (red) or (C) anti-CD19 (red) antibodies. Black scale bar = 100 μm. Supple. Fig. 6. Fluorescent IHC staining for FXIII-B & CDH11. Supplementary images for Fig. 3. Images of fluorescence staining with each antibody and low power field images. Black scale bar = 100 μm. Supple. Fig. 7A. Alteration of FXIII-B expression levels in passage culture of FLS. The expression levels of F13B in sy [file 41232_2022_252_MOESM1_ESM.pdf]
